# Neural Interfaces for Intracortical Recording: Requirements, Fabrication Methods, and Characteristics

**DOI:** 10.3389/fnins.2017.00665

**Published:** 2017-12-07

**Authors:** Katarzyna M. Szostak, Laszlo Grand, Timothy G. Constandinou

**Affiliations:** ^1^Next Generation Neural Interfaces Lab, Department of Electrical and Electronic Engineering, Centre for Bio-Inspired Technology, Imperial College London, London, United Kingdom; ^2^Department of Neurology and Neurosurgery, Johns Hopkins University, Baltimore, MD, United States

**Keywords:** neural interface, neural probe, intracortical, microelectrode, fabrication, implantable, microsystem

## Abstract

Implantable neural interfaces for central nervous system research have been designed with wire, polymer, or micromachining technologies over the past 70 years. Research on biocompatible materials, ideal probe shapes, and insertion methods has resulted in building more and more capable neural interfaces. Although the trend is promising, the long-term reliability of such devices has not yet met the required criteria for chronic human application. The performance of neural interfaces in chronic settings often degrades due to foreign body response to the implant that is initiated by the surgical procedure, and related to the probe structure, and material properties used in fabricating the neural interface. In this review, we identify the key requirements for neural interfaces for intracortical recording, describe the three different types of probes—microwire, micromachined, and polymer-based probes; their materials, fabrication methods, and discuss their characteristics and related challenges.

## Introduction

It is widely considered that our understanding of the human brain is science's final frontier. In recent years, we have witnessed a sustained and significant investment internationally in several initiatives toward this goal (Grillner et al., 2016). These fundamentally aim to improve our understanding; by developing new techniques and tools to understand function and disease models, but also to develop new therapies and devices.

**Figure 1:**
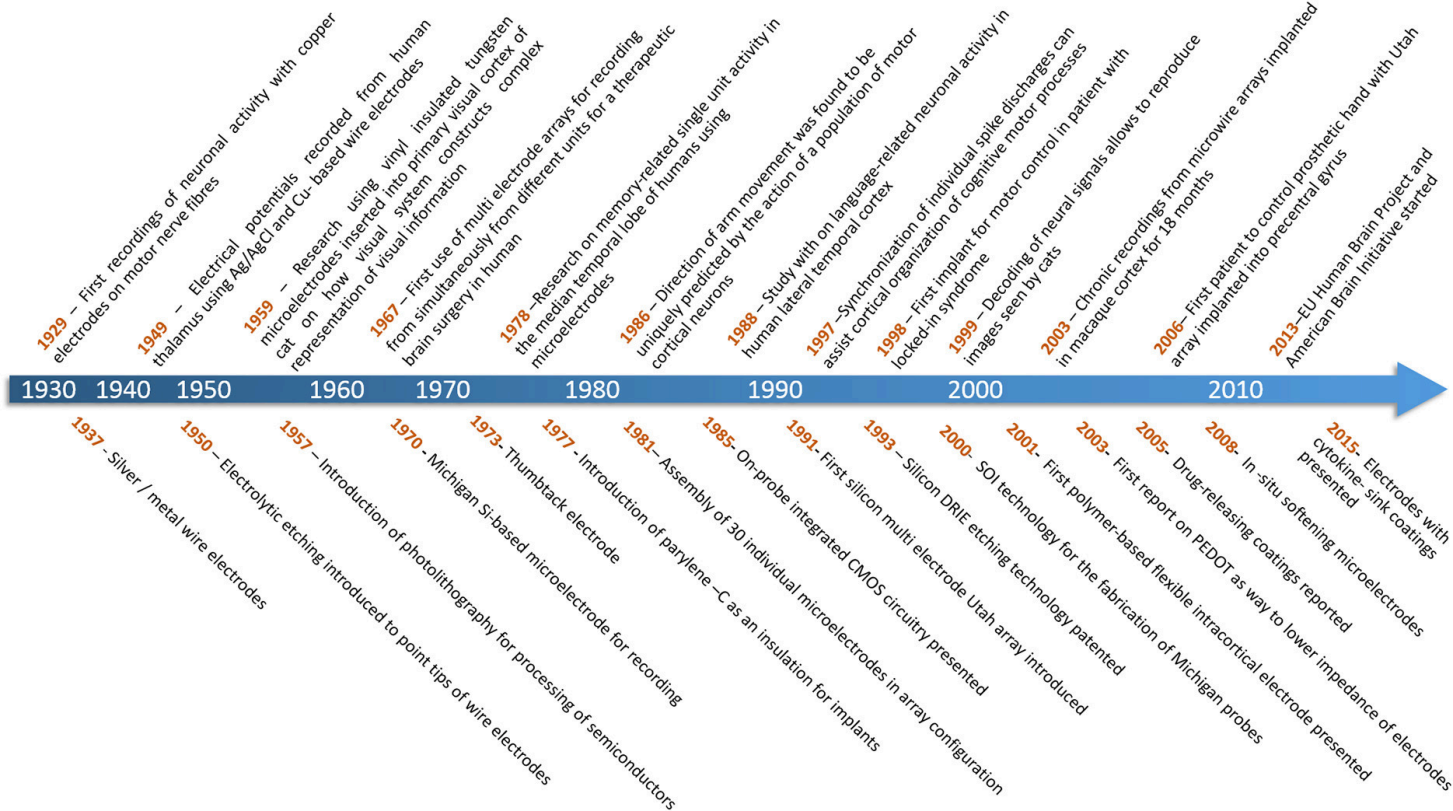
**Top:** Timescale of achievements in brain neuronal recording (Adrian and Bronk, 1929; Williams and Parsons-Smith, 1949; Hubel and Wiesel, 1959; Marg and Adams, 1967; Halgren et al., 1978; Georgopoulos et al., 1986; Ojemann et al., 1988; Riehle et al., 1997; Kennedy and Bakay, 1998; Stanley et al., 1999; Nicolelis et al., 2003; Hochberg et al., 2006). **Bottom:** Timescale of progress in technology of brain-computer interfaces (Rheinberger and Jasper, 1937; Grundfest et al., 1950; Jules, 1964; Wise et al., 1970; Bak and Salcman, 1974; Loeb et al., 1977; Krüger and Bach, 1981; Najafi et al., 1985; Campbell et al., 1991; Laermer and Schilp, 1996; Cheung et al., 2000; Rousche et al., 2001; Cui and Martin, 2003; Zhong et al., 2005; Capadona et al., 2008; Skousen et al., 2015).

For more than half a century neuroscientists have recorded the characteristic action potentials (spikes) generated by cortical neurons in order to understand how information is represented and transmitted through the nervous system (Hodgkin and Katz, 1949). Until recently, these experiments involved sampling small numbers of neurons over short sessions of a few hours, but with advances in microtechnologies, we can now record from hundreds of neurons over many weeks, months, or even years. The fact that such technology has enabled us to transition from experimental work on rodents, to monkeys, to human applications, in such a short time is a testament to the scientific and neurotechnology communities (Figure 1).

On the other hand, medical devices that interface to our nervous system exploit neuromodulation by intervening for pathological activity suppression, or by stimulating to bypass a dysfunctional element in the neural pathway, thus restoring normal functionality. For example, Deep Brain Stimulation (DBS) therapy has proven highly effective in the treatment of conditions such as Parkinson's (Deuschl et al., 2006), dystonia (Vidailhet et al., 2005), essential tremor (Grill et al., 2004), and more recently epilepsy (Boon et al., 2007). It is currently estimated that there are ~80,000 DBS implants in use today (Kestenbaum et al., 2015). Furthermore, sensory prostheses such as cochlear implants have had a significant impact on the quality of life of over 300,000 profoundly deaf individuals. This allows, for example, born deaf children to attend regular schools and develop speech naturally, and users in general to hear and interpret speech without lip reading. We are now also starting to see viable retinal implants that are becoming available to the blind.

Being able to control devices with our thoughts is a concept that has for long captured the imagination. Brain Machine Interfaces (BMIs) are devices that aim to do precisely this. This field is currently enjoying much interest in the scientific community with research stemming from the fundamentals of motor control to new electrode and device technologies. These efforts are now inspiring new translational efforts to develop such technology to communicate directly with the nervous system for therapeutic benefit. For example, neural signals from the motor cortex of paralyzed patients have been used to operate assistive devices such as computers and robotic prostheses (Hochberg et al., 2012).

To date however, neural interface technology has only had significant clinical impact in neuromodulation devices (e.g., DBS and cochlear implants). There have in fact only been a few examples, in humans, of effective BMI technology. This is because there are a number of unresolved biological and technological difficulties, which we have to overcome to achieve reliable long-term recording of the nervous system (Kozai et al., 2015). The fundamental challenge originates from the neural interface itself. This is typically an array of tiny conducting electrodes in contact with neural tissue; used to observe the electrical activity, and pass this onto an electronic device to record, decipher, and classify this to provide useful information. However, the body ultimately responds to any “foreign body” or implanted device in such a way as to isolate it, and protect the body from harm. This leads to scar tissue growth around any implanted devices resulting in the attenuation of any observed electrical activity making it challenging to distinguish from background noise.

Fortunately, there is a wealth of experience and knowledge in developing countless different neural interfaces for intracortical recording. The literature describes the physical structure and properties of the different designs, but also performance characteristics with experimental *in-vivo* recordings of both extracellular action potentials and local field potentials. Different types of electrodes are suited to different special resolution of signals, but additionally can exhibit different long-term performance. The materials, fabrication method, surface finish, geometry, biocompatibility, implantation method all play a role in this conundrum.

In this paper, we review the different types of neural interfaces that have been developed to date specifically for the application of intracortical recording. The paper is organized as follows: section Requirements for the Design of Intracortical Recording Electrodes defines the key requirements needed for next generation intracortical recording interfaces in terms of foreign body response, biocompatibility, mechanical, electrical properties; sections Wire-Based Arrays, Micromachined Microelectrodes, and Polymer Microelectrodes review the three key types of neural interfaces for intracortical recording: wire-based, micro-machined, and polymer-based respectively; section Assembly of Neural Interfaces outlines the key issues in subsequent assembly; and section Concluding Remarks concludes with a discussion.

## Requirements for the design of intracortical recording electrodes

Next generation neural interfaces for intracortical recording pose a number of challenges that are ever so more critical for this specific application. This section will outline relevant requirements including: (1) foreign body response; (2) biocompatibility (both relating to toxicity to the body, and corrosion due to body); (3) mechanical; and (4) electrical properties.

### Foreign body reaction

**Figure 2:**
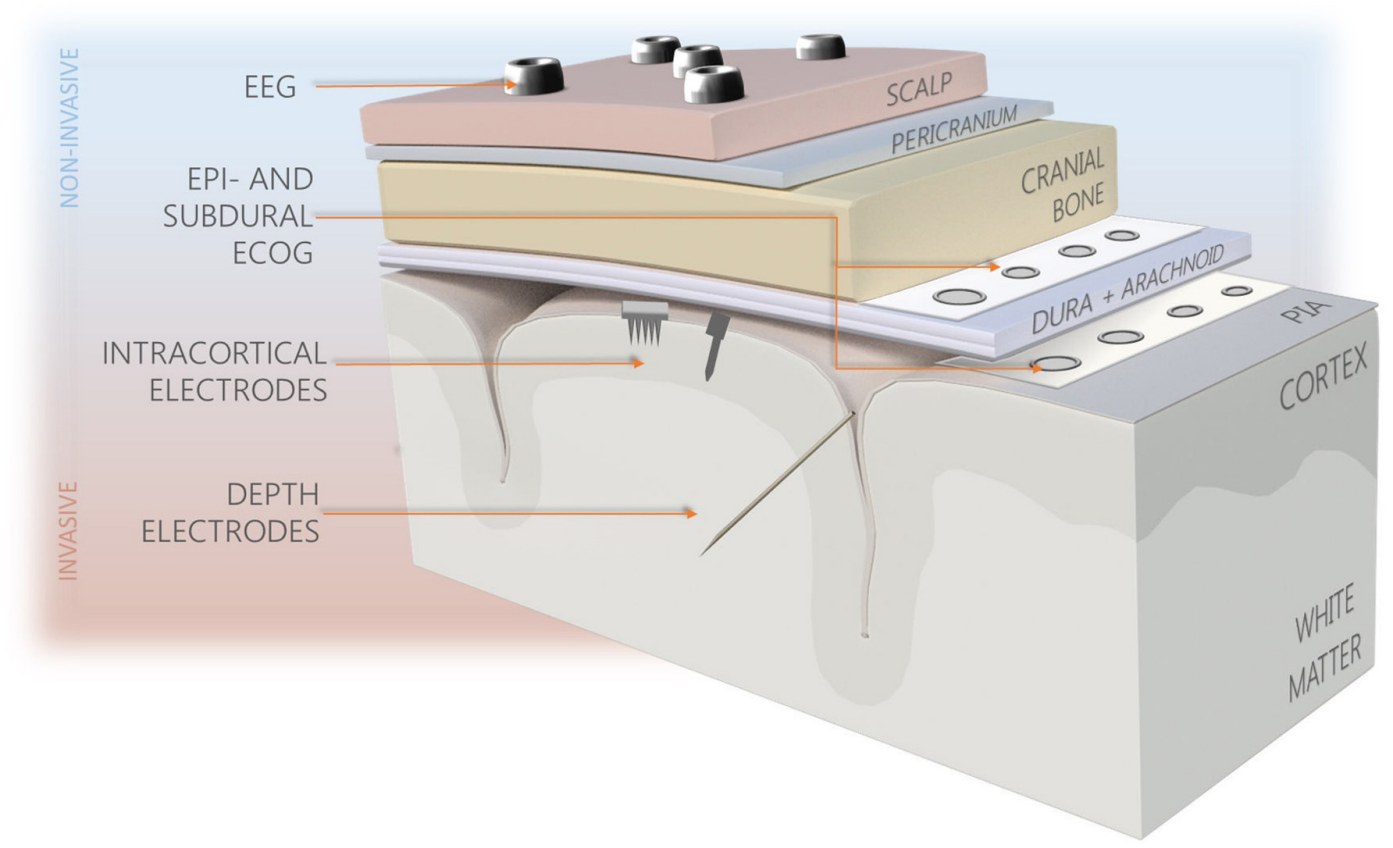
Types of brain interfacing electrodes and their location in the reference to the brain. Less invasive systems (blue background) provide recordings of lower resolution in comparison to intracortically-implanted electrodes.

Over the past 70 years, several different methodologies for neural recording varying in technology, recording resolution and invasiveness have been proposed, including electroencephalography (EEG), electrocorticography (ECoG) arrays, depth probes, and intracortical microelectrodes (See Figure 2). The latter provide better recording quality compared to less invasive technologies, as they are capable of recording different signal types (single, multi-unit activities, and local field potentials), and offer the best spatial and temporal resolutions. The significant disadvantage of intracortical probes' use is their limited longevity resulting from high degree of invasiveness and tendency of progressive worsening of recorded signal quality. To date, none of the probes developed are capable of completely overcoming the long-standing effects of foreign body reaction (FBR), despite variety of materials, shapes, and sizes used (Tresco and Winslow, 2011; Prodanov and Delbeke, 2016). It is believed that reactions occurring at the probe-tissue interface, implant characteristics, and the quality of initial implantation procedure are all influencing the performance consistency, however their mutual relations are not comprehensively explained.

FBR, body's self-defence mechanism, starts nearly immediately after insertion of the foreign object and evokes stream of events ultimately accountable for promoting electrode's deterioration and formation of scar tissue around implantation site (Grand et al., 2010; Kozai et al., 2015). Sudden rupture of vasculature around the implantation site leads to the release of blood constituents, activation of macrophages, phagocytosis, disruption of blood-brain barrier (BBB), and oxidative stress due to the presence of reactive oxygen and nitrogen species (Marin and Fernández, 2010; Kozai et al., 2015). At the onset of FBR, activated microglia proliferate to form lamellipodia-based encapsulation. Soon after, glial cells start moving their cell bodies toward injury site to form cellular sheath, which is further enriched by activated astrocytes. Over the time the encapsulation becomes denser populated with fibrotic and gliotic cells, what increases the distance between recording contacts and active neurons, and significantly limits ion and neurotransmitters flow (Polikov et al., 2005). Eventually it results in structural changes in cellular architecture, which may spread up to 150 μm from the implant. Entire process takes up to 2–3 months, with initial inflammation phase stabilizing after several weeks (Prasad et al., 2012; Sridharan et al., 2013). Over that time the quality of recordings from implanted microelectrodes degrades, what can be observed as a sudden impedance increase as measured by impedance spectroscopy (Ludwig et al., 2006; Leach et al., 2010).

Signal deterioration may be caused by prolonged reduction in neuronal counts. However, cell necrosis in implant vicinity progresses only at the early stage of FBR, thus it does not explain signal changes that occur later after initial surgery (Stensaas and Stensaas, 1976). Surface corrosion resulting from contact with saline-rich environment also can contribute toward recording failure, as it may produce potentially damaging oxidative or toxic species (Patrick et al., 2011; Prasad et al., 2012; Jorfi et al., 2014). One of the most common hypothesis suggests astrocyte hypertrophy as a main factor contributing to recording failure. It assumes that because of its extended thickness and high impedance, glial scar encapsulation isolates electrode physically and electrically from non-affected tissue strong enough to impede recorded signal and push active neurons away from recording zone. Yet, earlier studies indicate that glial proliferation does not progress in the absence of infection, suggesting inflammation and cellular reorganization resulting from persistent BBB damage are the dominant factors limiting long-term neuronal recordings (Saxena et al., 2013; Nolta et al., 2015). Products of activated microglial cells, such as pro-inflammatory cyto- and chemokines can lead to demyelination and cellular functionality degradation, what could also contribute toward failure (Giulian et al., 1994; Babcock et al., 2003; Winslow and Tresco, 2010). With varying degree, effects of FBR are present at implantation sites of all implantable electrodes. Limited neurodegeneration is observed similarly at the areas of iatrogenic stab wounds, suggesting that cellular architecture change is correlated with the extent of initial bleeding after surgery and is further amplified by the presence of implanted foreign body (Nolta et al., 2015).

### Biocompatibility

#### Toxicity

A neural interface that ideally minimizes the effects of FBR and works faultlessly for long, needs to satisfy many requirements regarding the architecture, size, shape, and material properties. Since the implant is in the direct contact with the tissue, it needs to be made of materials which can interface it without causing toxic, allergenic, or other harmful effects, are prone to the attacks of products of body metabolism and are able to perform their function over long time. The degree of implant biocompatibility is dependent on mechanical properties, chemical composition, microstructure, and surface characteristics (Williams, 2008). Studies on body response to thin wires of various metals implanted into feline cortex have shown the dissimilarity between reactions to different materials can be observed as early as week after initial surgery (Robinson and Johnson, 1961). Hence, materials employed for the construction of neural implant, that is substrate, encapsulation, and recording site material, should be characterized by good degree of biocompatibility, especially if aimed for chronic applications. Implant materials must also be prone to delamination, corrosion, breakage, and failure of interconnections. Moreover, none of the implanted materials should generate or leach another form of chemical products, such as oxidative species or solvents, as besides influencing recording capability they could contribute toward material degeneration (Patrick et al., 2011). To date in the formation of implantable neural interfaces, metals, glass, semiconductors, oxides, polymers, nanomaterials, and variety of hybrid materials have been employed. The most common materials present in the majority of implant designs as substrates, for recording sites and interconnections are metals. Gold, platinum, tungsten, iridium are found overall safe and are regularly used in bio-applications. Silver, silver/silver-chloride, pure iron, cobalt, palladium, and copper are considered toxic, as they provoke severe immune response. The choice of conductors must be done carefully, especially if it is an alloy, to avoid formation of galvanic cell structure, where less noble metal in pair will corrode in saline environment. In the newer neural probes designs, metallic layers are largely enhanced by conductive polymers (CPs). This is motivated by CPs biocompatibility, fast charge transfer, ability to be functionalized with nano- or drug-releasing structures, along with possibility to tune their conductivity by up to 15 orders of magnitude (Chen et al., 2013). As a result, recording sites of small geometry and low impedance can be formed, enabling good quality recordings. The most popular conductive polymers are Poly (3, 4-ethylenedioxythiophene) (PEDOT), polyaniline (PANI), and polypyrrole (PPy) which can be further doped to tune their properties with materials such as Poly(styrene sulfonate) (PSS) or paratoluene sulfonate (pTS). CPs are usually applied in the form of the blends with nanomaterials, hydrogels, or elastomers, as on their own they are characterized by elastic moduli in the range of gigapascals and have tendency to delaminate under prolonged electrical stimulation. More detailed information about materials considerations in neural electrodes are provided in Hassler et al. (2011), Fattahi et al. (2014), Merrill (2014), and Scholten and Meng (2015).

#### Mechanical properties

During surgical implantation, intracortical electrodes pierce layers of heterogeneous tissue—thin highly vascularized Pia and then neural tissue consisting of neurons, glial cells, myelin layers, venules, and capillaries. If not removed prior the procedure, also thick, fibrous Dura mater must be penetrated. Small tissue elements interlacing with each other form entwined structure that is easy to shear by much larger electrode tip. It is believed that sharp tips penetrate tissue with smaller displacement and pierce through capillaries causing less bleeding, unlike blunt tips that tear blood vessels' walls. Opposing theory suggests that sharp points cut tissue, whereas blunt tips push it away thus causing less trauma. Nonetheless, once implanted sharp points might persistently perforate vasculature, causing inflammation in the tip vicinity. Much research is focused on implantable microelectrodes' size reduction. It is believed that an electrode of the size comparable to neuronal body (12–15 μm) could lessen implantation trauma and reduce tissue's displacement and mechanical mismatch (Ludwig et al., 2011). Studies on relation between the implant's geometry and chronic performance have given mixed outcomes. On one side, it is suggested that smaller geometries are beneficial and lead to reduction in glial fibrillary acidic protein (GFAP) expression over time (Stice et al., 2007; Kozai et al., 2012); while older studies indicate size reduction is beneficiary only initially, and does not influence the formation of glial encapsulation (Szarowski et al., 2003). Smaller electrodes could provide higher selectivity and sensitivity for single unit recording; however, significant miniaturization of electrodes creates implantation problems and forces formation of even smaller recording sites.

Reduced dimensions and high aspect ratio of implantable electrodes usually effect in increased bendability, nonetheless typical probe materials are characterized by dramatically different mechanical properties comparing to neural tissue. Elastic modulus mismatch between brain and implant materials ranges between two and eight orders of magnitude. It is suggested that it causes probes to apply strain onto micro-moving brain, thus encouraging continuous local irritation (Lee H. et al., 2005). Pressure applied by stiff probes, along with lack of conformity with soft tissue may lead to the formation of gaps at the interface, which can fill with body fluids, thus shunting recording electrodes between adjacent sites, hindering recordings and moving electrode from the area of interest. Therefore, soft, flexible materials are preferred (Hassler et al., 2011; Fattahi et al., 2014; Nguyen et al., 2014; Xiang et al., 2016). Additionally, coating with soft hydrogels can be employed to decrease the mismatch at the initial phase after implantation. Hydrogels, including fibrin, alginate, hyaluronic acid, and polyethylene glycol, are advantageous in limiting mechanical mismatch and FBR. They are soft, moist, body-absorbable, and can be enriched with anti-inflammatory drugs, neural growth factors, or living cells to form biotic buffer layer between probe and brain tissue. The biggest drawback of hydrogels is lack of precise control over their thickness over time. Once implanted, hydrogel layers swell, putting pressure on adjacent tissue and increasing the distance between recording sites of electrodes. Some difficulties might also be encountered during implantation- if probes coated with hydrogels are too smooth; most likely, the layer will be removed upon attempt of insertion.

The significance of probes rigidity was proven in the studies comparing body response to implants differing only in directional flexibility, where astrocytic proliferation was considerably reduced in the axis in which electrode could follow tissue movements (Köhler et al., 2015). Probes made of flexible polymers implanted in the rat model, shown reduced inflammatory response and steadier BBB than stiff implants, particularly when compared at later time-points after surgery (Nguyen et al., 2014). Since inflammation results from the lack of mechanical compliance, probe's fixation mode becomes important factor amplifying the degree of irritation. Glial scarring and local neurodegeneration was observed to be less significant for the free-floating electrodes as compared to tethered probes. Furthermore, immunohistochemistry studies on fixation mode have proven that apart from changing the shape and enlarging implantation cavity, tethering flattens neurons at the probe interface and reorganizes adjacent neuronal architecture. Resulting gap fills with extracellular fluid averaging recorded signals (Thelin et al., 2011). Moreover, tethering usually requires forming transcranial and percutaneous connections, which effectively become an entry point for the infections. When tethering is inevitable, flexible cables are favored over rigidly fixing screws and adhesives, as they are better in accommodating relative brain movements while inducing up to two orders of magnitude smaller strain (Subbaroyan and Kipke, 2006). It is believed that ideal neural interfacing implant would be completely free of extra tethers and instead communicate via wireless link.

The shape and characteristics of the BMI electrode depends largely on technology, biological model, and target neuronal structures to record from. Generally, the geometry of electrodes should be tailored to the subject, as brain architecture differs substantially between humans, non-human primates, and rodents. Typically, probes consist of slender, few-millimeters long shaft on which recording electrodes are placed. The probe length and distribution of electrode sites along it depend on neuronal structures of interest i.e., when aiming to record from neurons located within brain sulci it is advantageous to have longer electrodes facing sideways along the probe length. It is desirable to fit multiple recording sites on single shank to allow registering depth profile of neuronal activity without necessity of displacing large volumes of tissue. In the majority of neural probes, the stem is either needle-like with conical tip or of the blade shape, though curved, and completely devoted of a rigid stem designs are also found. Histological, transcriptomic and electrophysiological studies on effects of probe geometry concluded that both in acute and chronic time points small cylindrical shapes evoke smaller body response that larger, planar structures (Karumbaiah et al., 2013). According to the studies comparing electrodes of the same size but different surface area, FBR was smaller in lattice structures, thus suggesting that proper implant's architecture can limit immune response.

Another factor influencing recordings and the extent of FBR is the quality and ease of implantation procedure. Neural interfaces need to be designed to mechanically withstand axial forces applied during implantation, without buckling or compressing tissue underneath. This is easier achievable for stiff, smooth implants with sharp tips, and small footprint. Probes of larger geometries, such as Utah electrode arrays, need to be implanted with an aid of pneumatic inserters, whereas implants of low Young's moduli are often temporarily stiffened with insertion aids and dissolvable coatings. It is also of great importance to ensure implant's shape allows for the explantation without generating more damage to the neuronal tissue affected.

#### Electrical properties

The relation between recording sites' size and impedance is inversely proportional, so that smaller electrodes are inherently more noisy, exhibit worse recording quality, and are less functional because of decreased maximum possible stimulating current. It is estimated that the resistance in the range of at least 40–150 kΩ is necessary to enable selectivity in detection of a single unit action potentials, whereas at electrode's impedance greater than 5 MΩ recording of neural signals is overpowered (Buzsáki, 2004; Prasad and Sanchez, 2012). One approach to address this is to retain recording sites small, but increase their effective geometrical surface area by roughening or functionalization with nanostructured materials such as platinum black, platinum grass, carbon nanotubes, and conductive polymers (Pigeon et al., 2003; Chung et al., 2015). For that purpose, plasma treatment along with electroplating, etching, laser, and electron beam patterning techniques are employed. In the studies comparing an effect of CF_4_ plasma on gold electrodes of polyimide-based neural probes, the roughening treatment resulted in 98% impedance drop and led to LFP recordings of two times greater signal amplitude (Chung et al., 2015). The attention must be given to induce roughness on nanometric level, as macroscopic roughness is undesirable from the insertion damage point of view (Edell et al., 1992). Surface structurization is also believed to promote cellular attachment and neuronal ingrowth because of its similarity to the nanoscale morphology of extracellular matrix environment and small surface energy (Silva, 2006).

**Figure 3:**
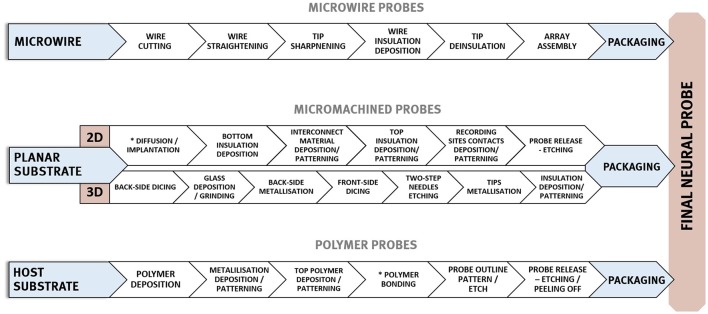
General, exemplary fabrication procedures employed in the formation of three main neural implants' types—micro wire based, micromachined silicon, and micromachined polymer-based probes.

A summary of the key properties required for electrodes used for intracortical recording is given in Table 1 and overview of fabrication processes applied in the manufacturing of three neural probes main technologies is shown in Figure 3.

**Table 1:** Summary of desirable properties and currently used parameters of electrodes for intracortical recording.

**Property**	**Value**	**Requirements**
Materials	Recording sites/Interconnections: Pt, Ir, Pt-Ir, Au, Iridium oxide, Polysilicon, W, Al Substrate/core: Si, Glass, Metal wires, Alumina, Polyimide, LCP, Parylene-C, SU-8, Silk Coatings: SiO2, SixNy, Glass, S-isonel, Teflon, PEDOT, PSS, PPy, CNTs, PEG, Laminin, Silk	Safe to use and able to reliably perform conducting Resistive to attacks of body fluids and products of metabolism Reliable and hermetic chronically
Young's modulus of implant	Polymers: 1 × 10^6^–1 × 10^9^ Pa Silicon: 130–185 × 1 × 10^9^ Pa Metals: >1 × 10^9^ Pa	As close as possible to the elastic modulus of brain tissue (0.1–1 × 10^3^ Pa) Allows for easy implantation without tissue dimpling Reduces movement-induced trauma
Average impedance range of electrode	<1 MΩ Typically: 20–150 kΩ (at 1 kHz)	Of low value to decrease noise Allows for recording of single unit activity Can be obtained with a large surface area (Cogan, 2008)
Dimensions of implant	Diameter: Preferably: <12 μm Typically ca 125 μm	As small as possible Allows decrease foreign body response effect Allows promote interface biocompatibility (Ludwig et al., 2011)
Power density of implant	<40–60 mW/cm^2^	Small to avoid heating up neural tissue more than 2°C (Wolf, 2008)
Signal to noise ratio	>5	As high as possible to appropriately differentiate shapes of spikes Not below 1.25, as it is then considered noise (Chapin and Nicolelis, 1999; Smith et al., 2002)
Recording site geometry	>50 μm	Allows to decrease impedance and improve recording selectivity As small as possible with high surface to area ratio. Larger for LFP recording (Nelson and Pouget, 2010)
Capacitance of electrode-tissue interface	150 pF–1.5 nF (Different depending on electrode area and surface roughness) (Harrison, 2008)	
Number of penetrating shanks per implant	>1–100/implant	Many shanks give space for more recording sites Of mechanical properties allowing for easy implantation, good tissue integration, and minimal tissue displacement during penetration Of possibly minimum volume to avoid extensive trauma
Density of recording sites per penetrating shank	>1–1,000	As many as possible to allow increase in spatial representation of recorded signal and to monitor several single neurons (Scholvin et al., 2016)

## Wire-based arrays

Microwire arrays (MWAs), or so-called wire electrodes are long established, commercially successful neuron-interfacing solutions. The history of using metal wire electrodes dates back to the early twentieth century with some initial studies on stimulation and recording with the use of silver probes (Rheinberger and Jasper, 1937). Nowadays MWAs are conventionally used in studies of neural activity in brains of rodents, non-human primates, mammals, and humans, especially in applications requiring stable long-term performance or interfacing deeper brain structures (Lehew and Nicolelis, 2008). Microwires have been used to record both single- and multi- unit activities, as well as LFPs over extended periods, for example, lasting over 9 months in the cerebral cortex of guinea pigs (Williams et al., 1999) over 18 and 84 months in motor cortex of macaques (Nicolelis et al., 2003), even seven years in monkeys (Krüger et al., 2010).

MWAs are based on insulated 10–200 μm diameter metal wires with an uninsulated tip used to observe the biopotential from neurons in vicinity. They are mainly constructed using methods of manual assembly from widely available components such as sockets, cables, and wires. This gives flexibility to tailor and adjust array's configuration parameters, such as effective wire length or spacing and allows for formation of simple, application-tailored, inexpensive designs that can be assembled without specialized equipment.

**Figure 4:**
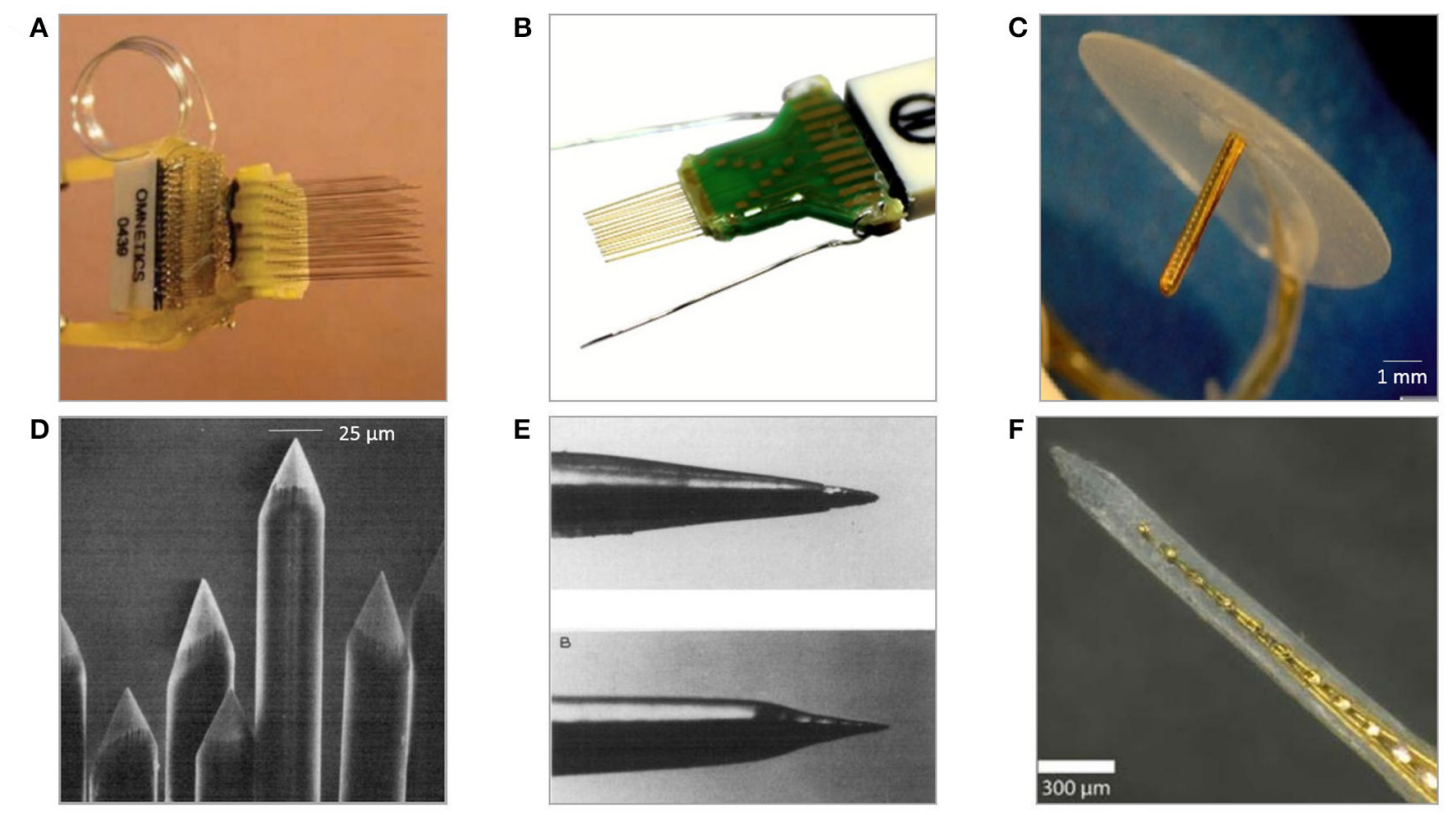
Examples of microwire-based technology neural electrodes **(A)** 64 channel, floating, discrete 8 × 8 microwire electrode array assembled into connector (Lehew and Nicolelis, 2008). **(B)** Tucker Davis' 32-channel layered polyimide-insulated tungsten wire array assembled onto custom PCB^1^. **(C)** Plexon's 24 channel linear Thumbtack microelectrode array (Ulbert et al., 2001)^2^. **(D)** Tips of insulated microwires sharpened mechanically on grinding wheels (Kaltenbach and Gerstein, 1986). **(E)** Various tips' shapes of eligiloy achieved by electrochemical sharpening of a microwire (Ashford et al., 1985). **(F)** University's of California 32-channel shank microelectrode array of gold microwires assembled within epoxy shank (Merlo et al., 2012).

There have been numerous assemblies proposed using wire electrodes of different shapes and sizes. These range from single wires and co-spun bundles up to organized multiple-lead arrays held together by printed circuit boards or connectors, with use of dental cement, methyl methacrylate, or polyethylene glycol. Significant scalability advancement of wire-based technology is so-called “thumbtack electrode” which was used to successfully record neuronal activity in neocortex of epileptic patients (Ulbert et al., 2001). It consists of polyimide-insulated platinum-iridium wires integrated within polyimide-epoxy shaft, and circular silicone sheet anchoring entire probe in place thanks to the surface tension (Figure 4C) (Hochberg et al., 2006).

The nature of fabrication and relatively manual assembly methods of microwire arrays lead to reproducibility challenges. Even when wires are processed as a single batch, there are some variations in the tip sizes, geometries, and lengths of uninsulated areas (Prasad et al., 2012). The limitation to the scalability of the number of recording sites is another disadvantage. More recording sites would allow for monitoring more single neurons, whereas microwires are constrained to only one electrode at the tip of each wire. Multiplexing recording instrumentation in microwire technology is possible only by using multiple leads, what results in an implant's size increase and substantial tissue displacement. Because of their high aspect ratio and tendency to bend during insertion, MWAs suffer from not being able to define, control, or even observe the precise locations of the recording tips. Prior to penetrating the cortex, microwires typically experience significant buckling and compress the underlying tissue (Ward et al., 2009). Once implanted, microwires have a tendency to splay, resulting in placement precision in the range of merely few millimeters (Matsuo et al., 2013).

Microwire—based electrodes are not free from recording failures and FBR effects. Several studies have shown MWAs to suffer from variations, disappearance or deterioration of recorded signal in the timeframes spanning from weeks to months post-implantation. Histological and structural evaluations of tissue after microwires' implantation suggest that they provoke immune response and glial formation, but in comparison with other microelectrode technologies induce only minor damage to local cellular environment (Freire et al., 2011). Analysis of tungsten microwires removed after 9-month implantation in a rats have shown substantial material degradation in the form of isolation cracks and delamination, along with considerably deep metal corrosion caused by electrochemical reactions (Prasad et al., 2012). Such extensive change in electrodes morphology results in increased recording surface area and electric leakages, possibly being the reason of recording problems. Generally, microwire recording failures are ascribed to the corrosion-inducing electrochemical reactions at the biotic-abiotic interface, insulation delamination, fractures, buckling, and FBR effects (Sankar et al., 2014; Sergi et al., 2016). Simplicity of MWAs is both a great advantage and source of challenges. Owing to the large size of sockets and co-components, downscaling of systems is limited and wires are difficult to connect to microelectronic packages. However, regardless to these challenges, MWAs to be in many cases, the technology of choice when it comes to chronic recordings (Nicolelis et al., 2003; Schwarz et al., 2014).

### Materials

The selection of metal for wire-based neural interfaces for recording is largely application-specific and depends on material stiffness, conductivity, corrosion resistance, and ease of processing. Most prevalent metallic wires which are considered safe for implantation and are characterized by favorable electrical properties, low impedance, and sufficient charge storage capacity, include tungsten, stainless steel, nichrome, iridium, platinum, and platinum-iridium alloys. Glass, Teflon, polyimide (PI), Parylene, and formvar are in turn standardly used metal insulation materials. The wire's size has a significant impact on the quality of the recording and implantation surgery. Wires of greater diameters are easier to insert because they do not bend upon contact with brain surface, however they cause larger tissue displacement enhancing resulting trauma (Thelin et al., 2011). The recording site is typically formed by terminating the wire end, so the size of the lead directly influences the impedance. Therefore, using thin wires results in recording sites of higher impedance, which if needed can be tuned by application of lower-impedance material (iridium oxide, platinum black, conductive polymers, nanomaterials) or roughening. Apart from metals, also carbon fibers have been reported to form wire-based arrays (Kozai et al., 2012). They are relatively stiff, have low electrical resistivity and a micrometer-scale footprint, thus ensuring low thermal noise and limited crosstalk. Because of having a very high Young's modulus, carbon fiber microelectrodes are easy to implant, and if coated with additional stiffeners can be placed a few millimeters deep into cortical tissue without causing significant injury.

### Fabrication methods

MWAs are usually handmade using relatively simple fabrication techniques. Main system components (wires, connectors) are acquired from commercial vendors, modified, and assembled into arrays. In general, the construction of microwire arrays consists of steps of wire shape modification (cutting, straightening), tip modification (sharpening, smoothening, functionalization), insulation (deposition, selective removal), and array assembly (braiding, gluing, bonding). If not post-processed, the performance of neuronal recording will be dependent on the quality and shape of a wire cut (Palmer, 1978). The smoothest cuts are obtained with lasers, sharp blades, and surgical scissors while conventional wire cutters tend to crush tips (Farina et al., 2013). Unless bought annealed, spooled wires can be straighten with the techniques of stretching, spinning under load, heating, or passing current through the wire under tension (Delgado, 1952; Tsai and Yen, 2003; Kim et al., 2007; Zhang et al., 2007). An alternative approach of manufacturing three-dimensional array of metal microelectrodes involves fixing gold wires onto the substrate using wire-bonding method, Parylene-C insulation and polishing to expose recording tips of blunt tips (Hetke et al., 1994).

#### Electrolytic etching

Electrochemical etching is the most predominantly used technique of alteration to create pointed, nanometric-diameter electrode tips. This enables processing automation and fabrication of tips that are gradually tapered, have various shapes or sharpness below micrometre range (Figure 4E) (Grundfest et al., 1950). Etching occurs at the interface between metal and electrolyte and is dependent on a number of parameters, including metal electrochemical properties, immersion depth, etchant concentration, or supplied power (Chang et al., 2012). Often the process is performed in a two-stage manner consisting of course electrolytic etching followed by electropolishing to smoothen and thin the wire (Lalanne et al., 2011). Alternatively, wires can be sharpened without the need of earlier insulation removal by mechanical polishing using rotating abrasive surfaces (Figure 4D). However, this technique results in larger tips and worse control over recording site size and impedance uniformity and requires superior alignment to obtain adequate symmetry of the taper (Kaltenbach and Gerstein, 1986).

#### Deposition of the insulating layer

Wires are coated with an insulation layer to ensure electrical isolation, mechanical integrity, and to increase biocompatibility with neural tissue. Dielectrics are mainly applied using electrochemical or casting methods, either on full length or locally below sharpened tip. The most popular insulation materials include Parylene- C, polyimide, Teflon, resins, and glasses, commonly applied by heat shrink, electrodeposition, physical vapor deposition (PVD), dip coating, CVD (Parylene), and fluidised–bed methods (Bartholomew, 1962; Loeb et al., 1977; Cooley and Vanderwolf, 1978; Perera and Mauretti, 2010). Coating with glass is usually performed by drawing wire through molten drop of material or by heat fusion with glass micro cylinder using micropipette pullers or heating rings (Wolbarsht et al., 1960; Levick, 1972; Sugiyama et al., 1994). Nonetheless, wires are generally purchased being already pre-coated because of the complexity of methods providing good quality pinhole-free insulation layers.

#### Tip deinsulation

To provide electrical connection to the neural tissue and lower impedance and electrical noise, the electrode tip must be exposed from under the insulation layer. There are variety of methods giving diverse level of control over the amount of removed insulator. Mechanical means of insulation removal include grinding (Kaltenbach and Gerstein, 1986), abrasion in air-borne stream of particles (Campbell et al., 1991), breaking (Jones et al., 1992), cutting (Cheung et al., 2000), and stripping (Burt, 1966; Verloop and Holsheimer, 1984; Jaeger et al., 1990; Jellema and Weijnen, 1991). However, they are likely to cause damage to the tip and remaining insulation. Glass insulation can be removed chemically in hydrofluoric acid. Other methods include burning off, melting, plasma etching combined with selective masking, or for certain organic materials—electron beam exposure (Skrzypek and Keller, 1975; Levy et al., 1986; Farina et al., 2013). Some polymers can be removed locally by taking advantage of their curing shrinkage, which automatically leads to the tip exposure (Green, 1958). The technique applicable for the removal of the majority of coating materials uses electric discharge. It is based on passing electric signal through the wire, so that the greatest current density occurs at the very tip of the probe, leading to discharge, insulation break, and exposure of underlying conductor (Loeb et al., 1977). Alternatively, insulation layers can be removed with precise control over the spot size using laser ablation. Proper choice of laser type (typically excimer lasers), wavelength, and pulse duration allows for accurate openings over tips without affecting electrode's metal core. However, laser ablation near the tip causes redeposition of carbon residue, which needs to be removed, as it may affect electrode's properties (Loeb et al., 1995).

#### Assembly

There are two main methodologies of MWAs assembly: discretely wired and layered approach. In both cases, spacing between electrodes is in the range of 200–1,000 μm (Lehew and Nicolelis, 2008). The discretely wired approach relies on handling and bonding wires to connectors independently. Custom-designed jigs and spacers are used to maintain desired clearances and overall shape of an array before leads are secured to connectors, which are then attached to signal processing units with flexible cables (Figure 4A). In the layered approach the spatial arrangement of wires within an array is determined by the layout of specially designed PCB, silicon or polymer preforms, within which microwires are placed and bonded (Figure 4B) (Jellema and Weijnen, 1991; Williams et al., 1999; Zhang et al., 2015). Variation of layered assembly technique involves threading microwires through pre-patterned acrylic mould, bonding loose ends to the PCB and then filling preform with epoxy glue forming a planar shank array (Figure 4F) (Merlo et al., 2012). Loose ends of wires are usually covered with epoxy or polyimide to avoid mechanical damage and protect from the external environment. Once attached to the connector, wires are secured with dental cements or epoxies to avoid undesired movement. The number of wires in the assembly depends on application and implantation's subject; nonetheless, arrays of as many as 96 wires were shown to work successfully for over a year *in vivo* recording signals from monkey's motor cortex (Wessberg et al., 2000).

## Micromachined microelectrodes

**Figure 5:**
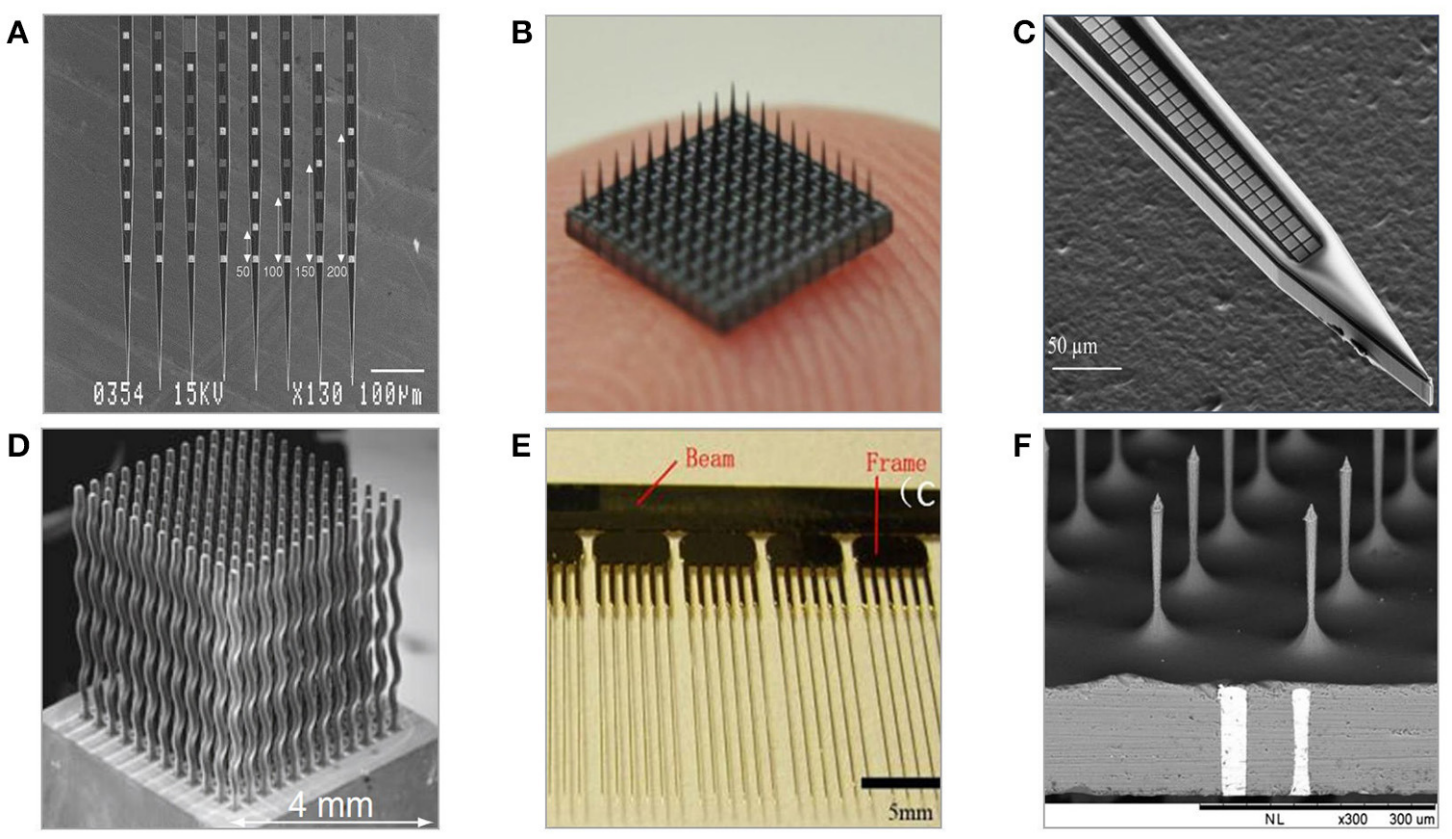
Examples of neural microelectrodes fabricated with micromachining methods on silicon substrate. **(A)** Michigan electrode—style 64-channel planar probes defined mainly with photolithography (Kindlundh et al., 2004). **(B)** 10 × 10 Utah electrode array fabricated from thick substrates by dicing and etching, size of array is roughly 4 × 4 mm (Yoo et al., 2012). **(C)** 1000-channel close-packed silicon microelectrode fabricated combining electron beam and standard photolithography (Scholvin et al., 2016). **(D)** Multineedle electrode array fabricated with wire electron discharge machining allowing for non 3D needle-shaping (Rakwal et al., 2009). **(E)** All-silicon wire electrodes fabricated by combination of wet and dry etching processes (Pei et al., 2014). **(F)** TSV-integrated silicon microneedle array (Chang et al., 2015).

The invention of photolithography and resulting progress in micromachining technologies triggered formation of the new generation of neural probes based on silicon (Jules, 1964). Wafer-scale microfabrication techniques enable freedom in designing 2D geometries, high precision with incomparable minimum feature sizes, integration with signal processing circuitry, and consistent large-scale fabrication. Micromachined microelectrodes form the largest, and the most diverse group of penetrating neural probes. Numerous designs of two and three-dimensional geometries, employing various materials and coatings have been proposed, and tested for periods up to a maximum of 81 and 300 weeks in non-human primates (Suner et al., 2005; Barrese et al., 2013). One of the key advantages of micromachined probes is the capability of having multiple recording sites, providing valuable information about spatial representation of neural activity and improving the identification of recorded signals. Generally, the majority of silicon-based neural microelectrodes designs are either examples or modifications of two-dimensional multisite Michigan electrode arrays (MMEAs) or three-dimensional multi-needle Utah electrode arrays (UEAs) (Figure 5).

### Michigan planar electrode arrays (MMEAs)

Historically, the first introduced silicon-based intracortical probe was the planar Michigan electrode array in the form of tapered, 15–50 μm-thick, 30–50 μm-wide silicon shank with multiple metal recording sites positioned along it (Wise et al., 1970). The fabrication flow of Michigan electrode involves series of photolithography steps combined with oxidation, metal deposition, lift-off, and etching steps finalized by passivation coating and device release (Wise et al., 2004). The geometry of electrodes is defined entirely by photolithography, thus giving unique design and customization possibilities. By adding more fabrication steps in the engineering process flow, probes can be integrated with IC circuits, microfluidic, and micromechanical structures. The length of probe's system depends on the application, and varies between a few millimeters up to few centimeters, with a typical length being ~5 mm (Hetke et al., 1994). The probe thickness is defined either by the SOI wafer's device layer, or by etching off the volume pre-defined by the depth of boron doping process. Micrometer-range dimensions benefit in a reduced tissue displacement, yet Michigan planar electrode arrays have been reported implanted and operational for short periods only because of recording loss problems (Vetter et al., 2004). Their planar recording sites are predominantly made of gold, platinum, or iridium, and can be modified by conductive polymers and nanomaterials. It is hypothesized that the 2D geometry of MMEAs-based recording electrodes is the reason for their failure in chronic recordings. This is because the geometry makes them more likely to be affected by the influence of distance-increasing body fluids and glial encapsulation. Insulation is provided by multiple layers of silicon nitride and silicon oxide and often protected with an extra polymer layer. Because of their aspect ratio and material rigidity, Michigan planar electrode arrays are prone to fracture. MMEAs are used mainly in rodent subjects for short-term independent and parallel recordings of LFPs, single and multi-unit activities from various cortex areas. Because of unique advantage of having multiple recording sites, MMEAs able to provide parallel recordings with great spatial representation.

Michigan planar electrodes can be assembled into 3D arrays by using silicon-based assembly platforms, to which probes are inserted and reflow-soldered using low temperature eutectic, wire-free ultrasonic, or flip chip bonding (Figure 5A) (Bai et al., 2000; Aarts et al., 2011; Cheng et al., 2013). Alternatively, microelectrode structures can be configured into array within custom flexible cables, which are then folded and fixed with an epoxy layer (Bai et al., 2000; John et al., 2011).

### Utah electrode arrays (UEAs)

Since their first appearance in early 1990s, Utah electrode arrays have grown to become the dominant reliable silicon-based neural microelectrodes in use, and so far, the only allowed for chronic human applications (Campbell et al., 1991). UEAs are bulk-micromachined, three-dimensional arrays of hundred, few millimeters–long needles having tip-recording sites coated with platinum or iridium oxide (Figure 5B). Overall form factor of the UEAs arrays is large and only certain aspects of their design geometry, such as electrode spacing, can be defined.

Fabrication of Utah electrode arrays includes sequence of dicing, glassing, etching, and deposition of metal and passivation layers (Jones et al., 1992). Electrodes are spaced regularly every 250–400 μm and isolated with a glass. Depending on the initial thickness of silicon substrate, the needles' height can range from several hundred microns up to a few millimeters, typically 1.5 mm. Because of the footprint, Utah electrode arrays are implanted in subjects of rather larger brain volume, such as cats, non-human primates, or humans. The placement surgery is complicated because of significant tissue dimpling, so pneumatic inserters-based, high-speed implantation procedures are employed. Tissue analysis performed after acute implantation of Utah electrode arrays usually reveal microhemorrhages and glial tissue encapsulation near the tips.

High density of recording sites within Utah electrode arrays allows for observing the activity of large neuronal populations with good resolution provided by tip-location of electrodes. UAEs are well-employed in the rehabilitative applications to both record and stimulate neurons to perform given task, such as control over prosthetic limb (Hochberg et al., 2006, 2012; Velliste et al., 2008). Studies focused on comparison of various electrodes *in vivo* shown that Utah electrode arrays obtained the charge capacity 13 times that of Michigan electrodes (mean of 10.4 and 0.8 mC/cm^2^, respectively), while generally averaged signal to noise ratio of Utah electrode arrays is in the range of 3–4.8 (Suner et al., 2005; Kelly et al., 2007). Alike other microelectrode technologies, the quality of signals recorded with Utah electrode arrays degrades with time. After 4 weeks from implantation less than a half of electrodes remains active, and even less after chronic periods (18% after 52 weeks) (Fattahi et al., 2014). The reasons for recording loss are seen in aforementioned vascular damage, FBR, interconnection failures, size, and rigidity of probes. In comparison to MMEAs, Utah electrode arrays perform better in chronic applications what it is believed to be caused by location of recording electrodes at the tip of needles rather than at the side of the shanks.

### Materials

The majority of micromachined neural electrodes are made on either semiconductor, glass, or metal substrates and employ materials standardly utilized in CMOS and MEMS industries, such as oxides, nitrides, polymer, and metal layers. Following demanding ISO10993 tests common MEMS materials including Si, silicon thermal oxide, polysilicon, silicon nitride, titanium, SU-8 and silicon carbide, are considered biocompatible and non-irritant (Kotzar et al., 2002).

**Figure 6:**
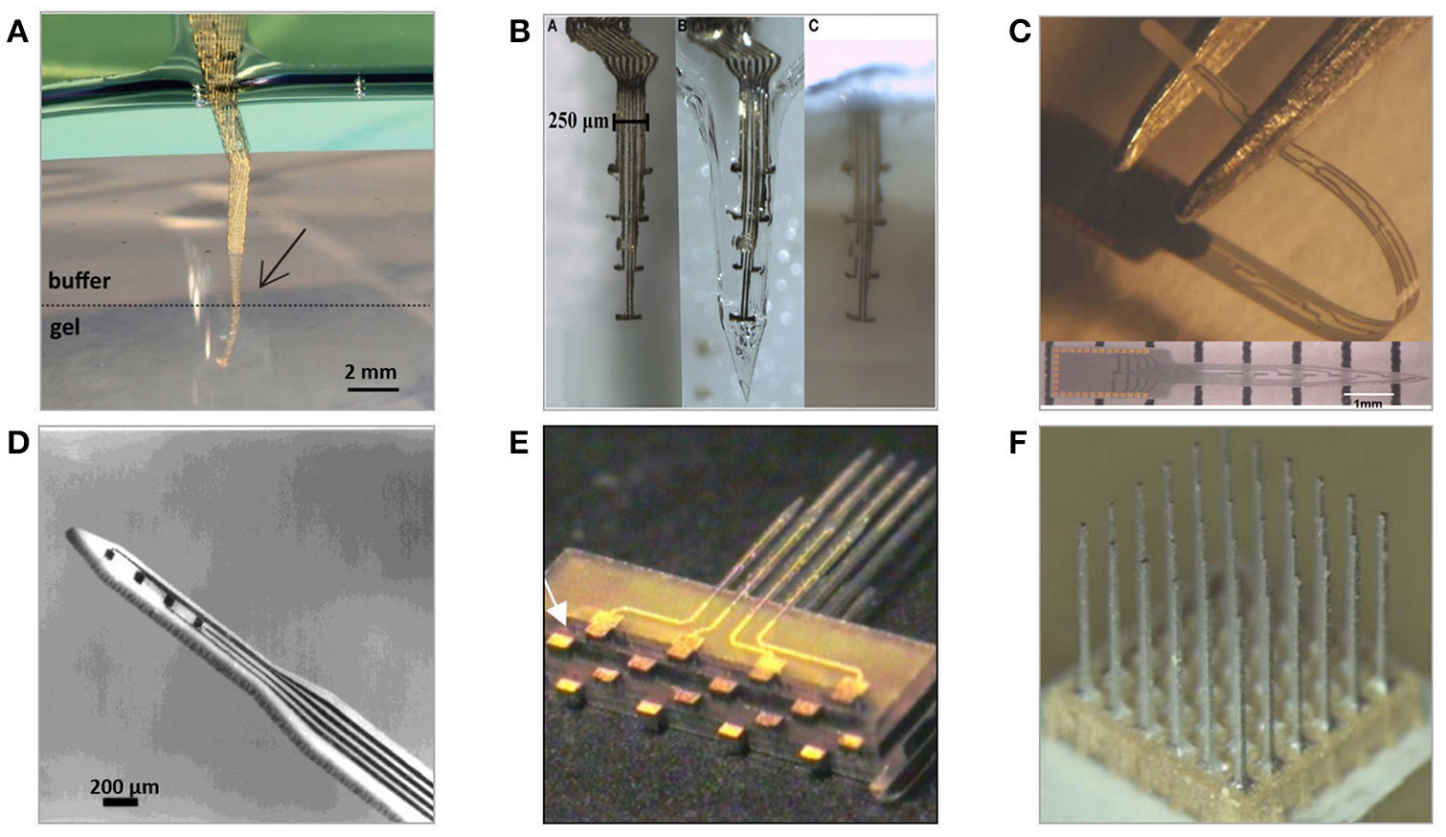
Neural microelectrodes fabricated from various materials with the use of micromachining methods **(A)** Three-dimensional, flexible macroporous thin layer metal microelectrode (Xie et al., 2015) **(B)** Highly flexible metal layer electrodes with implantation-enabling dissolvable gelatine matrix (Agorelius et al., 2015) **(C)** Diamond-based planar microelectrode (Chan et al., 2009) **(D)** Ceramic-based planar microelectrode (Burmeister et al., 2000) **(E)** Multilayer planar glass-based microelectrodes array (Lee et al., 2009) **(F)** Three-dimensional, aluminum-based 6 × 6 multineedle metal microelectrode (Goncalves et al., 2014).

Fabrication of Si-based neural electrodes is carried on either a silicon or silicon-on-insulator (SOI) wafer. Using a SOI wafer has an advantage in the fabrication of planar electrode arrays, as the buried oxide layer depth automatically determines the shank thickness and provides the possibility for double side lithography (increasing total number of recording sites per implant) (Cheung et al., 2000; Norlin et al., 2002). Utah arrays are manufactured using a non-typical, thick silicon wafers to allow for the creation of fine microneedles by dicing. Attempts have been made in structuring bulk metal blocks of stainless steel or aluminum into Utah-type microelectrode array geometries using correspondingly the same processing steps (Figure 6F) (Pigeon et al., 2003; Peixoto et al., 2012; Goncalves et al., 2014). However, resulting needles were of an average uniformity and required extra coating with non-oxidizing conductive and isolation layers.

Selecting substrates of proper crystallographic orientation allows taking advantage of crystal-plane dependent anisotropic etching, which influences the shape of resulting structures (Bassous, 1978). The use of borosilicate glass as a structural substrate for implantable probes isolation is believed to be beneficial in improving isolation capabilities thus decreasing possible losses and crosstalk (Figure 6E) (Lee et al., 2009; Lin et al., 2009). Other neural probe substrate materials include CVD–silicon carbide, gallium phosphide, as well as diamond and alumina ceramic substrates (Figure 6) (Moxon et al., 2004; Chan et al., 2009; Suyatin et al., 2013; Saddow et al., 2016). Electrical insulation is typically provided by multilayers of silicon oxide and silicon nitride and often extra-protected by Parylene-C, Liquid Crystal Polymer (LCP), or polyimide polymer coatings.

### Fabrication methods

As mentioned previously, micromachined microelectrodes utilize wafer-based fabrication methods commonly used in CMOS and MEMS industries. This section briefly outlines the key process steps for patterning and adding/removing conducting or insulating layers.

#### Patterning

The capability of transferring geometric shapes onto substrates by the means of lithographic techniques is essential to form two and three-dimensional structures. Photolithography and its derivatives largely determine the fabrication procedure of neural electrodes, thereby enabling early and precise definition of probes' features, such as size, geometry, spacing, and interconnections layout to fit targeted neuronal areas. Instead of standard photolithography, more accurate electron-beam lithography can be used for transferring patterns of very densely spaced features over small space like planar electrode's shank (Figure 5C) (Scholvin et al., 2016). Because of the three dimensionality and high aspect ratio of UEAs, achieving good uniformity during resist coating and development is difficult, what makes photolithography challenging. To address this, masking can be done by piercing a sheet of aluminum foil through needles (Bhandari et al., 2009b). In Utah electrode arrays, standard photolithography is used for the patterning of bonding pads at the backside of each needle (Jones et al., 1992). Direct laser writing paired with deep reactive ion etching (RIE) can be used instead of photolithography to selectively open windows in the passivation over the recording spots along planar silicon probe shank, allowing fitting neuronal structures of an interest with even bigger probe design flexibility (Kindlundh et al., 2004).

#### Sawing

Silicon dicing is a fundamental technique in the fabrication of UEAs deciding on overall arrangement of arrays. Both sides of the wafers are cut using dicing saws equipped with ultra-thin diamond blades capable of cutting narrow (down to 50–70 μm) kerfs to outline location of the needles and grooves to be filled with insulating glass. Most common for this application are resin-bond and nickel-alloy diamond blades (Ghane-Motlagh and Sawan, 2014). In the standard UEAs configuration, two orthogonal sets of 13 grooves are cut on each side with the aim of obtaining typical arrays of 10x10 columns, creating an extra frame of columns surrounding array that is proven helpful in improving uniformity of further etching step (Bhandari et al., 2010b). Initial backside dicing outlines the base of each electrode and the gap between electrodes; second is deep cut on the front-side to define needles height. Dicing can be modified by the introduction of an extra step of gradient-depth dicing, taking place before cutting column shapes, which enables the fabrication of diverse-height slanted or convoluted arrays (Branner et al., 2001). For Utah electrode arrays instead of sawing, photolithography followed by deep reactive ion etching (DRIE) can also be used (Bhandari et al., 2011).

#### Grinding and polishing

Methods to decrease the total thickness of the substrate or deposited layers are performed using either purely mechanical, or combination of mechanical, and chemical techniques. For the fabrication of UEAs, after deposition of insulation, grinding, and polishing is performed until smooth finish is obtained (Bhandari et al., 2010a). This is done to allow an access to the back contacts and remove excess glass layer, which would otherwise significantly increase the volume of an array. To create a smooth surface of thickness variation less than 1 mm, polishing under low force is done using alumina slurries and wheels equipped with sets of various grit-size emery papers (Solzbacher, 2006). Grinding and polishing are widely used in fabrication of glass-based electrodes. The first is employed to remove glass excess from mould and latter to improve adhesion on thin metal layers to ground glass (Lee et al., 2009; Lin et al., 2009). Chemical-mechanical polishing provides superior flatness quality and was shown capable of thinning silicon neural sensing microsystems to expose metallic conductor fill of through-silicon-via structures, and in planarization of Parylene-C over silicon dioxide in the processing of multi-sided polymer-based implantable microelectrode arrays (Seymour et al., 2011; Huang et al., 2016). Fine polishing of three-dimensional structures can be achieved by electropolishing, to smoothen tips of wire microelectrodes after electrochemical etching, or to polish the surfaces of electrode shanks shaped by wire electrical discharge machining (Pigeon et al., 2003; Lalanne et al., 2011).

#### Etching

Etching is one of the fundamental manufacturing steps in both MEMS and semiconductor technology that in combination with lithographic techniques enables localized, controlled removal of material. Correct combination of diverse etch methodologies gives a tool to define or modify shape of probes as well as to change their surface morphology.

Isotropic etching of silicon is a chemical process obtained in acidic baths in the presence of strong oxidiser such as mixture of hydrofluoric and nitric acid diluted in either water or acetic acid (HNA etch). Typical 1:19 HF:HNO_3_ is used in processing of Utah electrode arrays to transform diced silicon columns into sharp needles by two stage procedure of column-thinning dynamic etching combined with tip-pointing static etching (Bhandari et al., 2010b). HNA isotropic etching can be employed to etch buried microfluidic channels in Michigan planar electrodes and smoothen of dicing and WEDM-induced roughness (Cheung et al., 2003; Chen C. H. et al., 2009; Grand et al., 2011).

Anisotropic etching properties of silicon reveal under exposure to alkaline solutions, and are expressed by various etch rates for areas with different crystallography or doping level. So-called silicon etch-stop typically realized in ethylamine pyrocatechol (EDP) or tetramethylammonium hydroxide (TMAH) is adapted for defining thickness and outline as well as to release planar microelectrodes (Najafi et al., 1990; Lin and Pisano, 1999; Yao et al., 2007). This technique is based on attribute of some etchants to dissolve highly p-doped material with marginally or considerably smaller rate, thus by earlier heavy boron doping controlled termination of etching is possible. Drawbacks of EDP-etch stop use include CMOS-incompatibility and doping depth limit, which is preventing construction of electrodes thicker than 15 μm (Lin and Pisano, 1999). With the knowledge of crystallography and appropriate mask design, etching in potassium hydroxide (KOH) or CMOS-compatible tetramethylammonium hydroxide may be used to define geometry of thick shanks (Xiao-Hong et al., 2007).

When silicon is electrochemically etched in solutions containing hydrofluoric acid, it results in the formation of porous silicon, the porosity of which depends on material's initial doping level. It can be utilized in neural probes as an on-probe biomolecular filtering element, easily removable sacrificial layer or as a polymer-probe's stiffening backbone, which degrades once implanted (Bell and Wise, 1998; Hajj-Hassan et al., 2012; Sun et al., 2016). The combination of dual side photolithography, anisotropic, and isotropic wet etching was used in fabrication of silicon-based wire electrodes having fully circular cross-section (Figure 5E) (Pei et al., 2014).

Because of superior process control, dry etching methods have largely replaced wet etching. In the construction of intracortical electrodes, dry etching methods are more prevalent for MMEAs fabrication especially when done on SOI substrates. In that case, boron doping is avoided, and instead two-step reactive etching is employed on the both sides of a substrate (Norlin et al., 2002). The first etch defines the probe outline at the top device layer and the backside etch releases structures out of wafer. The application of dry etching of planar silicon probes can be used to create a lattice structure which facilities cellular regrowth (Wise et al., 2008). With a low-temperature silicon oxide mask, dry etching allows to be fabricate Michigan electrodes with thicknesses in the 5–90 μm range on non-doped standard silicon wafers (Yoon et al., 2000). XeF_2_ dry etching can be applied to create random porous dense silicon microstructures that supports cell adhesion, increases adhesion of subsequently deposited layers, and improves impedance (Zhang et al., 2014). Combining DRIE and wet isotropic etching of silicon with SU-8 polymer integration was employed during fabrication of three dimensional silicon electrodes rooted in dielectric polymer substrate (Pemba et al., 2013). Three-dimensional formation of glass neural microprobe can be done by reflowing material into DRIE pre-patterned silicon wafer mould (Lee et al., 2009; Lin et al., 2009). In this case, the implant shape and thickness are the reflection of profile and depth of etch.

Recently, focused ion beam—based ion milling and laser ablation were used to fabricate multiple recording sites along Utah array shafts turning them into multi-side recording probe (Shandhi et al., 2015).

A summary of various techniques for etching and their application specifically to the fabrication of microelectrodes is given in Table 2.

**Table 2:** Comparison of etching techniques employed for the fabrication of electrodes for neural recording.

	**Wet chemical etching**		**Dry etching**	**Electrolytic etching**
	**Isotropic**	**Anisotropic**		
Methods	Wet chemical baths, vapors Chemical	Wet chemical baths, Vapors Chemical	Plasma etching Gas phase etching Reactive ion etching Deep reactive ion etching Physical, chemical-physical	Wet chemical baths with the application of electric potential Electrochemical
Properties	Uniform material removal Rounded shapes High etch rates	Material removal with different rates in areas of different crystallographic orientation or doping level Great process control Etch stop possibility Etch rates dependent on temperature and concentration Undercutting	Isotropic, Anisotropic Deep etching Minor broadening and undercutting CMOS compatible Repeatable Possibility to form structures with vertical sidewalls and small features	Combination of electrical and chemical reactions causing anodic dissolution of metals under the application of voltage between material and counter electrode
Materials/Solutions	Silicon: HF, HF/HNO_3_/CH_3_COOH Silicon nitride: H_3_PO_4_ Tungsten: HF /HNO_3_ Aluminum: H_2_O/HF; HCl/HNO_3_ /HF Platinum, Iridium: HCl /HNO_3_	Silicon: KOH, NaOH, EDP, TMAH	Silicon: XeF_2_; RIE- CF4, SF_6_; DRIE- SF_6_/C_4_F_8_ Polymers: O_2_, F_2_ – based plasmas	Silicon: solutions containing HF Tungsten: KOH, NaOH, Platinum, Iridium: CaCl_2_, HCl, NaCl, KCl, NaOH, KCN
Application in fabrication of neural electrodes	Two stage etching in the formation of Utah electrode arrays: pillar thinning dynamic etch and static- sharpening etch (Bhandari et al., 2010a) Smoothening of roughness induced during dicing and WEDM in Utah electrode arrays (Rakwal et al., 2009) Etching of buried microfluidic channels in planar probes (Cheung et al., 2003) Edges smoothening (Grand et al., 2011) Releasing electrodes from the substrate (Chen C. H. et al., 2009)	Thickness and shape definition of planar probes (Najafi et al., 1990; Lin and Pisano, 1999; Yao et al., 2007) Releasing planar electrodes from the wafer (Edell et al., 1992) Shaping electrodes accordingly to crystallographic planes (Xiao-Hong et al., 2007)	Thickness and outline definition of SOI-based planar electrodes (Norlin et al., 2002) Main removal technique in polymer-based neural interfaces (Kim and Meng, 2015) Release of planar electrodes from the wafer (Suzuki et al., 2003; Fernández et al., 2009; Chung et al., 2015) Formation of macroporous and lattice structures promoting neuronal ingrowth (Wise et al., 2008) Roughening of probes' surface (Chen et al., 2014; Zhang et al., 2014) Deinsulation of recording sites (Yao et al., 2006)	Sharpening metal wires for microwire electrodes using DC voltage for sharp hyperbolical tips, or AC-voltage for larger, angled conical shapes up to tens of nanometers (Grundfest et al., 1950; Chang et al., 2012) Electropolishing to smoothen surfaces and thin wire-based probes (Lalanne et al., 2011) Formation of porous silicon used as on-probe biomolecular filtering element, sacrificial layer, or dissolvable stiffening material (Bell and Wise, 1998; Hajj-Hassan et al., 2012; Sun et al., 2016)

#### Electrical discharge machining

Electrical discharge machining (μ-WEDM, EDM) is a technique using electrical discharge from a metal microwire in dielectric media to melt and vaporize a conductive material (Ho and Newman, 2003). It is an interesting alternative to sawing and etching used in the construction of the Utah electrode arrays. Micro wire EDM is capable of producing structures of the aspect ratios reaching 1:100 and creating three dimensional geometries with enhanced shape complexity (Figure 5D) (Fofonoff et al., 2004). Electrodes produced by discharge machining can be made of silicon, stainless steel, or platinum and exhibit flexure-type, spring-like, tapered, or variable length-profiles, which are normally not achievable with methods of standard microfabrication (Ho and Newman, 2003; Fofonoff et al., 2004; Rakwal et al., 2009; Tathireddy et al., 2009). The possibility of fabricating a wide range of shapes may be advantageous to create geometries aiming to increase lateral compliance between electrodes and nervous tissue. Limitations of μWEDM include extensive surface roughening and incapability of machining very thin features, as electric discharge-caused heat dissipation would induce bending (Bhandari et al., 2011). Hence, electrodes must be fabricated thicker, subsequently thinned and smoothened in isotropic wet etching or electropolishing steps.

#### Material deposition

To coat probes with different materials, a variety of physical and chemical deposition methods can be applied, such as thermal oxidation, electrodeposition, casting, physical, and chemical vapor deposition. Selectivity of layer coverage is provided by post-deposition material removal in processes of wet and dry etching, laser ablation, or lift-off techniques. The most straightforward are casting methods, but these can be used only for materials applied from liquid phase, such as insulating polymers and resins, and they do not provide exceptional control over layer characteristics. Electrochemical deposition methods offer coating with a wide range of thicknesses directly onto chosen areas, sufficient design flexibility and good layer quality, but require conductive surfaces and may result in large edge- build-ups (Madou, 2002; Du et al., 2009). Electrodeposition remains the main deposition method for complex hybrid layers containing organic materials, like mixes of conductive polymers, nanotubes, hydrogels, and bioactive compounds, which when applied onto electrode sites boost their electrical properties and influence surface morphology (Yoon et al., 2007). Physical vapor deposition methods produce high quality inorganic layers and might be used with shadow masks, whereas chemical vapor deposition methods are probably offering the largest flexibility in deposition but are complex and require high temperature processing (Seshan, 2002).

##### Depositing conducting layers

Conductors form the main group of materials to be selectively deposited onto the surface of neural probes to create interconnections, bonding pads and recording sites (Merrill, 2014). They should be of low impedance, chemically inert, and adhere strongly to the substrate. Conductors conventionally applied in the fabrication of neural electrodes include gold, platinum, iridium, and iridium oxide (Negi et al., 2010; Merrill, 2014). Direct deposition of certain metals on semiconductors or polymeric films might be problematic; therefore, it is often preceded by pre-deposition of adhesion and seed layers of well-adherent metals such as titanium or chromium. Techniques of sputtering and evaporation are the most common way of depositing thin conformal metal layers onto neural probes. Sputtering produces films of smaller grain sizes and somewhat better step coverage and adhesion than evaporation, nonetheless overall quality of deposited films is comparable (Adamov et al., 1974). By altering coating conditions, layers can be deposited porous or of high roughness which was shown potentially advantageous in increasing site's surface area or promoting cell adhesion (Cogan, 2008). As an alternative to metal, interconnection lines can be made of low-pressure chemical vapor deposited (LPCVD) polysilicon, which has been used as surface-roughening underlayer (Paik and Park, 2003).

##### Depositing dielectric layers

Dielectric layers provide probes with electric insulation and barrier from corrosive, saline-rich body environment. They should be conformal, biocompatible, defect-free, and of low dielectric constant. Deposition of thick or stiff layers may cause bending of probe structures, which limits safe implantation. For that purpose, isolation materials and their thicknesses should be chosen so that coefficients of thermal expansions are matched and residual mechanical stresses minimized (Yao et al., 2007). Silicon oxide and silicon nitride combinations usually deposited with CVD methods typically insulate silicon-based probes. Nonetheless, in the long term they were shown to be unstable and slowly degrade in saline environment (Cogan et al., 2003; Hsu et al., 2009). UEAs are generally difficult to be coated uniformly with standard deposition methods because of their complex three-dimensional geometry. Historically, the first approach to insulate electrodes in Utah array consisted of aluminum thermomigration to create deep p+ regions within n-type substrate to form opposing p-n junctions (Campbell et al., 1990). This solution had poor yield and was causing leakage and asymmetry of impedances between electrodes. Nowadays glass insulation is used instead (Bhandari et al., 2009a). Alternatively, electrodes can be electrically isolated and encapsulated within medical-grade epoxy resin removed from kerfs by dicing (Goncalves et al., 2015). In general, standard inorganic passivation layers are not sufficient to provide appropriate level of protection from body environment, thus supplementary encapsulation with polymers such as Parylene-C or polyimide is applied (Loeb et al., 1977; Hassler et al., 2011).

##### Depositing coating layers and surface modification

The last group are the layers that change electrodes' properties by decreasing impedance, altering biological response, improving mechanical conformity, or changing the surface morphology. These materials usually consist of nanostructured layers, conductive polymers, carbon nanotubes, or various bioactive films and can be applied on the majority of the neural interfacing probes (Fattahi et al., 2014). Electrochemical methods are dominant in the surface modification processing because they are rapid, inexpensive, and allow forming coatings of controllable composition. Electrodeposited coatings of conductive polymers (CP) are widely used to improve electrical properties of metal electrode sites (Zhang et al., 2014). Out of several CPs PEDOT and its modifications along with polypyrrole are the most widespread (Green et al., 2008). Widely deposited to decrease impedance and increase surface area of electrodes is platinum black, which requires special electrodeposition methods, such as pulsed plating or sonic plating to increase its durability (Desai et al., 2010; Zhang et al., 2015). Carbon nanotubes application causes significant increase in surface area and hence improves charge storage capacity and injection limit. Carbon nanotubes can be applied either by casting when suspended within other materials, directly by CVD, or by electrodeposition, which for neural applications is was shown to produce coatings of good adhesion, non-toxic, and stable properties (Fattahi et al., 2014).

## Polymer microelectrodes

Two of the key factors limiting quality of neural recordings of silicon-based and wire microelectrode arrays are their size and mechanical mismatch with brain (Nguyen et al., 2014). Using polymers can potentially overcome the disadvantages of stiff materials, and create conformal contact with soft, non-uniform neural tissue. Having lower Young's modulus and being stretchable, polymers do not provoke large strain on the tissue, hence limiting the secondary inflammation (Varner et al., 2016).

However, implantation of soft and flexible structures into the brain is challenging as the precision and depth of implantation is compromised. Attempts to overcome this problem are done with the construction of various insertion aids in the forms of removable stiff-backbone stiffeners, additional layers of dissolvable materials or by piercing the tissue with other instruments prior to the implant placement (Takeuchi et al., 2005; Felix et al., 2013; Castagnola, 2014; Barz et al., 2015).

**Figure 7:**
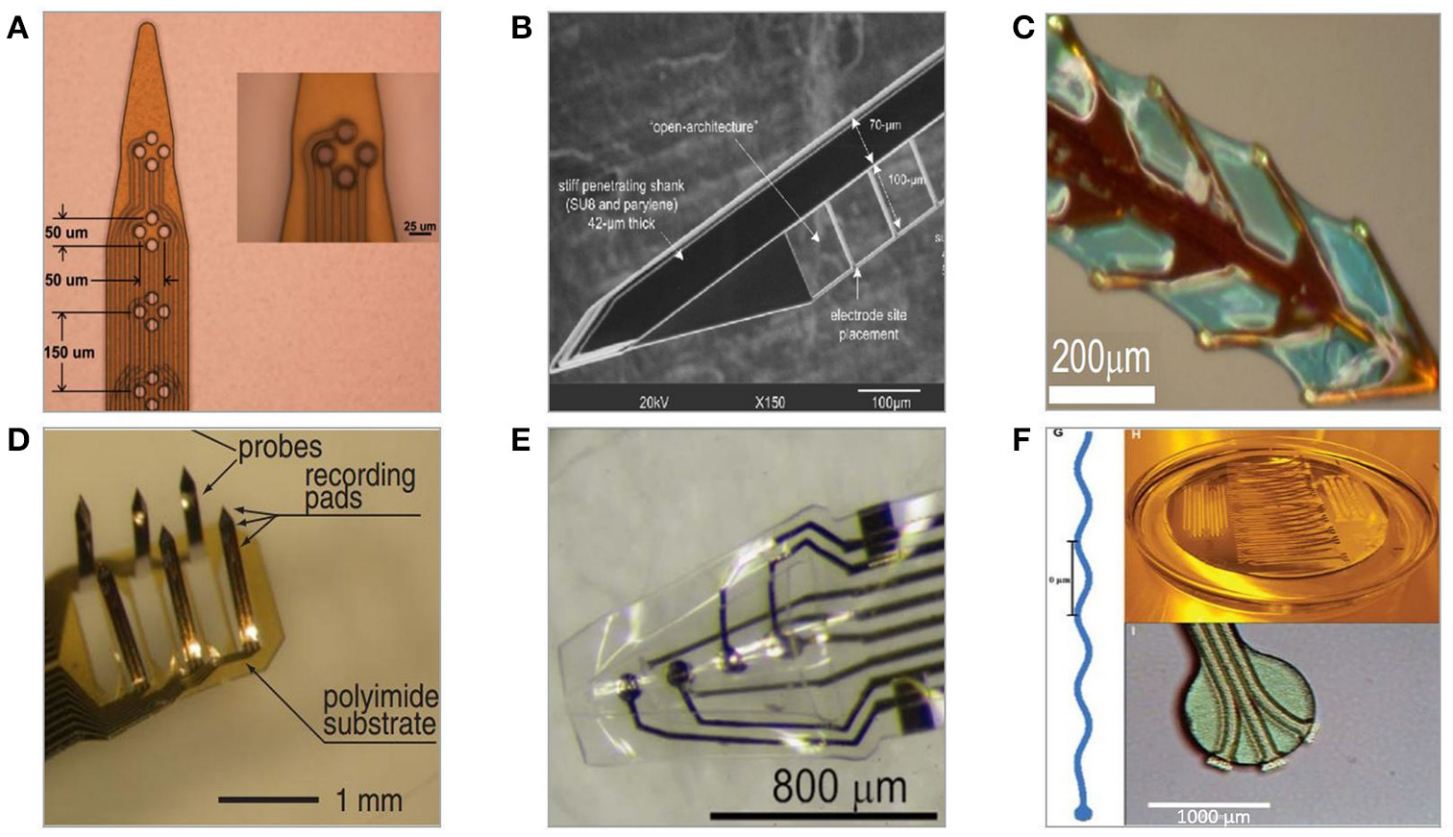
Polymer-based neural microelectrodes formed with a use of microfabrication techniques and host substrates. **(A)** Flexible polyimide-based planar multisite shank electrode (Mercanzini et al., 2008). **(B)** Parylene-C/SU-8-based flexible microelectrode with thin lateral arms allowing for mechanical mismatch compensation (Seymour and Kipke, 2006). **(C)** Polyimide-based fishbone-shaped microelectrode (Wu et al., 2011). **(D)** Polyimide-based three dimensional multichannel electrode (Takeuchi et al., 2003). **(E)** Three-dimensional thermoformed Parylene-C-based cone polymer sheath electrode (Kuo et al., 2013). **(F)** Parylene-C-based sinusoidal electrode (Sohal et al., 2014).

From the design point of view, most polymer-based neural implants resemble the solutions fabricated in silicon, such as slender shank-shapes with multiple metallic recording sites along the length of the probe (Figure 7A). However, owing to polymers mechanical properties and fabrication possibilities, some more unique geometries such as mesh, fishbone, or sinusoid are feasible (Figure 7) (Wu et al., 2011; Sohal et al., 2014; Xie et al., 2015).

Representative processing of polymers for the fabrication of neural interfaces differs between various implant types, but usually includes steps of polymer deposition, patterning, metal integration, and possibly bonding. Using polymer technology for the creation of neural interfaces benefits in the CMOS-compatibility, flexibility in material combinations and possibility of functionalization with additional components such as anti-inflammatory drugs, nanoparticles, or carbon nanotubes (Fattahi et al., 2014). Moreover, polymer technology allows an access to the large selection of polymer-specific processing techniques, such as photo patterning, thermoforming, or soft lithography (Qin et al., 2010). The problem with processing polymers as structural materials is their inability to withstand high temperatures that limits the use of some of the common microfabrication methods, i.e., certain deposition techniques (Kim and Meng, 2015). Certain microfabrication processes, such as thermoforming, electrochemical cleaning, or heat treatment can alter polymer electrodes' electrical properties, thus the methods should be chosen so that the final implant meets required electrical parameters (Hara et al., 2015). Polymers are characterized with water uptake that causes delamination, mechanical, and adhesion problems *in vivo* and can change probe's electrical performance over time (Jorfi et al., 2014). Because of that, flexible electrode arrays are limited to acute and short-term neural recordings. Thus far, the most of the research involving polymer-based intracortical electrodes in animal studies was focused on implants insertion behavior, however some acute and short-term recordings in rats' cortex were performed (Takeuchi et al., 2003; Seymour et al., 2011). Polyimide-based sheath electrodes implanted in rats motor cortex for the period up to 28 days, exhibited impedance within kΩ-range (Kim et al., 2013), whereas silk-backed Parylene electrodes of MΩ-impedance range picked up neuronal activity from the same area for 6 weeks (Wu et al., 2015). Polyimide-based electrodes of similar, MΩ-impedance range, successfully recorded mice motor cortex neuronal activity for 60 days (Cheung et al., 2007).

Currently, the technology of polymer-based neural recording probes is under constant development with the new designs being presented continually. Recently, polymer and polymer-metal multimodal probes for electrical recording and optical stimulations were fabricated in a process of thermal drawing of fiber from multi-polymer preform (Lu et al., 2014). Alternatively, polymers can be used to form flexible regions on standard silicon electrodes thus creating hybrid silicon-polymer probes (Schander et al., 2016).

### Fabrication methods

The choice of polymers for the neural applications depends on whether they are intended to be used as a substrate, encapsulation or functional element of neural implant (Hassler et al., 2011). During formation of polymer-based probes, to provide stiff base and ensure compatibility with processing equipment, silicon, or glass wafers are used as a temporary host substrates. Out of many polymers introduced for neural implants, a few gained the most attention due to their inertness, CMOS-compatibility and possibility to be used as both a substrate and encapsulation, including polyimide, Parylene-C, LCP, benzocyclobutene (BCB), and SU-8 (Lin et al., 2002; Hassler et al., 2011; Gwon et al., 2016). They are characterized by chemical inertness, low moisture permeability, low dielectric constant, and can be functionalized with organic agents. Additionally, SU-8 is popular for use in high aspect ratio structures and soft-lithography moulds (Lorenz et al., 1998; del Campo and Greiner, 2007). Having the same polymer as a substrate and encapsulation, simplifies overall fabrication as apart from deposition of conductive materials, no additional coatings are required and no mechanical mismatch between bottom layers and passivation occurs. Similar to the silicon equivalents, recording sites of polymer—based neural electrodes are made mainly of thin metal films of gold or platinum and improved by CPs.

#### Polymer deposition

Several polymers can be spin-coated to be as thin as 1 μm, among them polyimide, BCB, PDMS, and SU-8. The latter can be spun to reach as thick as 500 μm (Lin et al., 2002; Huang and Fu, 2007). To avoid probe bending due to in-built stresses, it is important to ensure that both bottom and top polymer layers are of similar thicknesses (Cammarata, 1994). Following deposition, certain polymers like PI or BCB need to undergo thermal curing treatment to remove solvents, and complete cross-linking process. This step influences the quality of the final film, thus its parameters must be well-controlled. Otherwise, when many polymer layers are deposited, it might result in shank bending, like in case of BCB-based probe (Lee K. et al., 2005). Polyimide and SU-8 in the form of cured thin sheets for lamination are also obtainable. Parylene-C does not require curing as it is applied via room temperature CVD, forming layers that are highly conformal, pinhole-free, of thicknesses ranging from hundreds of nanometers to several micrometers (Fortin and Lu, 2004). The ability to deposit Parylene-C at room temperature allows applying films onto surfaces structured with thermally fragile materials. This enables the creation of flexible neural probes integrating fluidic micro channels, which can be temporarily filled with PEG to increase their stiffness during implantation (Takeuchi et al., 2005).

#### Patterning

When using photo-patternable polymers such as polyimides, BCB or SU-8, material properties can be altered via exposure to light in standard photolithography processes. After initial soft cure and UV exposure, pattern can be developed and whole layer cured (Lin et al., 2002). In the case of Parylene-C and non-photopatternable polyimides, an additional layer of photoresist must be spun over cured polymer, which is then selectively removed in RIE or laser ablation processes. Polymers can be micro-patterned in soft lithography replication techniques (Qin et al., 2010; Wu et al., 2011). Droplet backside exposure is a method enabling the fabrication of millimeter-high slanted SU-8 microneedle/optrode arrays in single UV-exposure of polymer droplet locked to hydrophilized PDMS substrate (Kwon et al., 2014). The process of “drawing lithography” takes advantage of the change of SU-8 viscosity at different temperatures to construct 3D flexible array of millimeters-high microneedles on flexible, polyimide mesh substrate (Xiang et al., 2016). Some polymers have the ability to become thermoformed. Moulding two patterned sheets of Parylene- C over tapered stainless steel wire template can be employed to form conical microchannel promoting neuronal ingrowth in novel sheath-electrodes (Figure 7E) (Kim et al., 2013; Kuo et al., 2013; Hara et al., 2015). However, thermal treatment of Parylene-C induces changes in its crystallinity, and results in increased stiffness, tensile strength, and impedance of the probe (Kim et al., 2014; Hara et al., 2015). Most polymers can be patterned in a process of laser ablation in high power light Nd: YAG, CO_2_, or excimer lasers. It is possible to obtain the sub-micron minimum ablated feature size, however it is typically in the range of several micrometers (Lippert, 2010). Laser ablation affects the area around patterned features and can result in carbon residue deposition or creation of scorch marks. In that case, plasma etching after ablation is necessary to avoid redeposit-caused degradation of electrodes electrical properties (Terasawa et al., 2006; Lippert, 2010). On the other hand, laser structuring gives unique design freedom as it does not require additional masks or photoresists. Laser ablation is particularly advantageous in removal of polymeric insulation layers from the three-dimensional recording tips in MWAs and UEAs, where other methods do not provide comparable accuracy.

#### Metallization

Metallization of polymers is performed with physical vapor and electroless deposition methods (Ulrich et al., 1999; Cheung et al., 2007). Once the area is metallized with thin film, it can be further coated by electrodeposition to achieve thicker layers. Difficulties accounted during deposition of metals onto polymers are poor adhesion and mismatch in thermal and mechanical properties, causing metal films to crack when probes are subjected to high temperatures or significant strain (McClain et al., 2011). Both can be improved with the use of adhesion promoting layers and pre-roughening of polymer surface (Nakamura et al., 1996; Mercanzini et al., 2008; Castagnola, 2014). Rather than using physical deposition methods, metals can be applied a foils, which are integrated within a polymer sandwich by lamination. Such a metallization is less flexible but more robust (Schuettler et al., 2005). Highly conformal planar metal arrays of intracortical probes can be achieved via thin layer deposition, or thick laser-patterned foil integration, within polymers or dissolvable materials such as polyethylene glycol, gelatine or silk fibroin, which under exposure to body fluids expose single metal tracks (Figure 6B) (Agorelius et al., 2015; Wu et al., 2015). Well-engineered design and fabrication techniques of patterning, thermal metal evaporation, and SU-8 insulation were shown in the formation of three-dimensional macroporous lattice metal electrode that integrates signal-enhancing nanowire field effect transistors within its geometry (Xie et al., 2015).

#### Material removal

Owing to chemical inertness, wet etching of developed and cured polymer films is a challenging task. Polyimide is not dissolved in standard solvents and just few solutions were reported to attack its layers (piranha, strong acids, dedicated-etchants, hot bases, and to some extent resist strippers) (Ghosh and Mittal, 1996). Correspondingly, wet etching of Parylene and SU-8 faces similar problems as the former is only possible with a small selection of high-temperature solutions, and the latter can only be wet etched in molten salt baths, which is too aggressive for well-controlled fabrication of structures aimed for bio-applications (Dentinger et al., 2002; Kim and Meng, 2016). The best way to etch the majority of polymers is to use the conventional methods of dry etching in oxygen or fluorine plasmas (Kim and Meng, 2015). Beneficial to promote cellular or metallic adhesion on polymer surface is higher surface roughness, which can be achieved by RIE (Chen et al., 2014). Final release of polymer-based implants from the host substrates can be performed by dry etching of probe outlines followed by either mechanical peeling off the substrate, or by removal of interlayer of sacrificial material (Cheung et al., 2007; Chung et al., 2015). Thin layers of metals or oxides are often used for that purpose, as well as other polymers such as PI Kapton film (Fernández et al., 2009).

#### Bonding

Owing to the polyimide flexibility, structures made of it can be folded, or bent to become three-dimensional geometries. One way of achieving it relies on having the backside of probe electroplated with thick layer of nickel, which lifts shanks of fabricated neural electrodes up when exposed to externally applied magnetic field, resulting in formation of freestanding out-of-plane flexible neural electrodes (Figure 7D) (Suzuki et al., 2003). To create more complicated or microfluidics-integrating geometries, top and bottom polymer layers can be processed on separate host wafers and joined in subsequent bonding processes (Ziegler et al., 2006). Thermal bonding is commonly used to join stacks of same or dissimilar polymers. It relies on applying pressure onto the polymer stack, followed by vacuum treatment at the temperature between transition and melting temperatures of given polymer. It was successfully applied in the neural electrodes integrating microfluidic channels made of SU-8 and Parylene (Ziegler et al., 2006).

## Assembly of neural interfaces

To create standalone neural recording system, sensing units consisting of arrays of probes need to be connected with an external world and encapsulated within a hermetic package (Jiang and Zhou, 2009). Essentially, there are two strategies of hermetic packaging, one consist of direct deposition of encapsulation material over the implant and other involves having elements enclosed within moisture-protected cavity. Materials and techniques for packaging and assembly should not worsen probes' electrical and mechanical properties, degrade in the body environment or complicate implantation process. Instead, they ought to provide hermetic seal, be lightweight and of small form factor. Prior to implantation, the final package must withstand sterilization processes that engage harsh thermal, radiation, or chemical conditions (Lerouge and Simmons, 2012). Despite high level of miniaturization achieved for penetrating probes, complete neural interfacing systems remain large because of the limitations and the availability of assembly and packaging methodologies. Flexible cables, interposers, connectors, and various bonds are responsible for providing mechanical and electrical connectivity (Hetke et al., 1994; Yao et al., 2007; Ling et al., 2011). Methods of sealing and joining neural recording system elements include flip chip, epoxy adhesive, and wire bonding. The majority of silicon and polymer probes have the advantage of monolithic integration with flexible ribbon cables, which are defined in the same processing scheme parallel to the sensing units (Pang et al., 2005; Yao et al., 2007). This approach prevents conducting traces from being exposed to body environment. Flexible cables purchased from commercial vendors, or fabricated in the separate run, can be either wire bonded or reflow-soldered to the substrates (Bjune et al., 2015). Planar microelectrode arrays can be die-attached with epoxy glues, wire-bonded to the customized PCB boards and attached to the sockets for the connection with head-stage units using cables (Chen Y. Y. et al., 2009). Utah-type arrays are usually backside bonded to flexible wire bundles or cables, or flip-chip bonded either to interposer substrates, or to the active CMOS substrates (Patterson et al., 2004). The volume of the whole system can be significantly decreased by employing single-chip integration scheme. This can be achieved by the same substrate-integration and through-silicon-vias connectivity (Chou et al., 2014; Huang et al., 2014; Chang et al., 2015). Full system integration was achieved for UEAs integrated with data processing units, power supply, and telemetry link in multi-level hybrid assembly containing flip chip bonding, reflow soldering, and adhesive bonding to form compact standalone package (Kim et al., 2009).

## Concluding remarks

In this article, the summary of requirements and microfabrication methods used for intracortical recording microelectrodes, and their impact on the final design is presented. Developing fully implantable electrode, which would be capable of stable chronic neuronal recordings without harmful influence on the tissue, would contribute to revolutionizing our current understanding of the brain. The availability of such a technology would provide opportunities for new clinically viable neural prosthesis applications. The choice of probes' manufacturing technology depends on the construction material and desirable precision, which are in turn related to the preferred type of signals to be recorded.

Currently existing solutions rely primarily on the machining methods adapted from semiconductor and microsystem industries (Table 3). This is because semiconductor micromachining–based technologies give the best precision and design flexibility, and allow for straightforward integration of electronics. However, owing to the material's stiffness such probes were shown to cause severe tissue displacement, and cause long-term reliability problems. Moreover, not all of the materials potentially appropriate for neural recording applications can undergo CMOS/MEMS micromachining, because of lack of the planarity or incompatibility with processes' conditions. Most of the processes and equipment used in the fabrication of neural electrodes are designed with an aim of processing the surface of large, stiff, planar semiconductor wafers to form integrated electronic circuits–very different from what is required from small three-dimensional implants. Because of the technological limits of current microfabrication methods, significant scaling down of standard electrode arrays is no longer feasible and new paradigms must be found. Especially in the field of interconnection and packaging technology, there is still much room for improvements. Nowadays, potentially beneficial, small probes must be connected with large, stiff connectors and cables that apply tethering force on neural tissue. Instead, it would be advantageous for electrodes to communicate fully wirelessly, but that will require some work to overcome problems of tissue attenuation and local heating. Other techniques of precision mechanics, laser structuring and wire discharge machining are beneficial economically, but lack high throughput and exact precision.

**Table 3:** Comparison of microelectrode technologies for intracortical recording.

	**Wire–based probes**	**Micromachined substrate—based probes**	**Polymer-based probes**
Materials	Substrate: Thin metal wires of 10–200 μm diameter Conductors: W, Pt, Pt-Ir, Au, Stainless Steels, Elgiloy, Nichrome, Carbon Fibre, Conductive Polymer Coatings Insulators: PTFE, Parylene-C, polyimide, PMMA, epoxy, glass, S-isonel	Substrate: Silicon, Silicon-on-insulator, Semiconductor, Glass Wafers, Alumina Conductors: Au, Pt, Pt-Ir, Metal Silicides, Polysilicon, Conductive Polymers, Iridium Oxide, Chromium Insulators: Silicon Oxynitride, Glass, Parylene-C, Polyimide, Silicones	Substrate: Host silicon/glass wafer, polyimide, BCB, SU-8, PDMS, Parylene, LCP Conductors: Cr/Au Insulators: same as the substrate
Probes	Discrete-wired arrays (Palmer, 1978; Williams et al., 1999; Tsai and Yen, 2003) Layered-arrays (Zhang et al., 2007; Merlo et al., 2012) Wire bundles (Kubie, 1984) Single wires (Salcman and Bak, 1973)	Michigan electrode (Wise et al., 1970; Bai et al., 2000; Aarts et al., 2011; Cheng et al., 2013) Utah electrode array (Campbell et al., 1991; Jones et al., 1992; Bhandari et al., 2009b) Planar multisite electrodes (Chan et al., 2009; Lee et al., 2009; Suyatin et al., 2013; Saddow et al., 2016) Three-dimensional needle arrays (Pigeon et al., 2003; Peixoto et al., 2012; Goncalves et al., 2014) Multi-shank, multisite designs (Shandhi et al., 2015)	Planar flexible probes (Cheung et al., 2007; Wu et al., 2011; Lu et al., 2014; Sohal et al., 2014; Xie et al., 2015) Three dimensional thermoformed probes (Kuo et al., 2013) Thermally drawn polymer fibre probes (Lu et al., 2014)
Methods	Handmade assembly Electrolytic etching Ultrasonic bonding	CMOS/MEMS micromachining Wire electrical discharge Flip-chip bonding	CMOS/MEMS micromachining Laser structuring Moulding Thermoforming Flip chip, thermal bonding
Recordings	Duration: Acute, Chronic 18 months (Nicolelis et al., 2003) up to 7 years (Krüger et al., 2010) Subjects: humans (Halgren et al., 1978; Ulbert et al., 2001), rodents (Cooley and Vanderwolf, 1978; Williams et al., 1999; Zhang et al., 2007), cats (Rheinberger and Jasper, 1937; Salcman and Bak, 1973; Palmer, 1978) primates (Schwarz et al., 2014)	Duration: Acute, Chronic up to 81 and 300 weeks with Utah electrode array (Rousche and Normann, 1998; Suner et al., 2005; Barrese et al., 2013) or 18 weeks with Michigan electrode (Vetter et al., 2004) Subjects: humans (Hochberg et al., 2006), rodents (Yoon et al., 2000; Vetter et al., 2004), primates (Suner et al., 2005)	Duration: Acute (Patrick et al., 2011; Seymour et al., 2011), short up to 8 weeks (Cheung et al., 2007; Kim et al., 2013; Wu et al., 2015) Subjects: rodents (Cheung et al., 2007)
Comments	Long lasting Well-established Small dimensions Possibility for access to brain deep structures Technology reproducibility and implantation accuracy problems Simple and non-expensive	Freedom of design with great dimension control Stiff, easy to implant Cause damage and large tissue displacement Possibility for microfluidic integration Possibility for on-chip electronic circuitry integration	Non – hermetic Flexible and conformal with tissue Possibility for microfluidic integration Compatible with standard micromachining methods Not compatible with high temperature processes Implantation problems due to flexibility Allow to use a number of material-specific fabrication techniques

Inversely proportional relation between the technology accuracy/repeatability and chronic performance of fabricated devices can be observed. The best long-lasting recording performances are achieved with microwire electrodes. It might be caused by several reasons, including small footprint, overall simplicity of design, and material integrity. However, this technology does not give much room for increasing recording sites density and has limited possibility of wireless communication and electronics integration. As compared to micromachined solutions, the lack of common standards and methods of improving repeatability in microwire technology impedes deep analysis of manufacturing influence on chronic recording. Nonetheless, MWAs remain the number one choice where recording stability is the main concern. FBR is present, but less extent than for micromachined probes. On the other hand, some wire materials may not fully fit biocompatibility requirements because of the possibility of corrosion and release of toxic species, like in case of tungsten. Moreover, wire electrodes are difficult to scale down and are often difficult to implant, as the control over implantation depth and location is limited due to their bendability. Because of the shape of the microwires, local patterning for the control over the size of recording tip is troublesome.

On the other hand, silicon-based technologies are the most accurate and repeatable of them all, but already very well-exploited. Silicon technology is not envisaged to bring any significant breakthroughs in brain implants' performance unless new-engineered materials and novel hybrid methods are used. The most accurate and well-controlled processes and techniques are used in the fabrication of MMEAs, which because of their planarity can directly adapt technologies from CMOS industry. This results in the formation of the probes with the largest number of electrodes so far, which are highly customizable, but perform poorly chronically. Fabrication of Utah electrode arrays also utilizes micro-engineering techniques, but not as directly–it requires certain adaptations and creative manufacturing approaches. Nonetheless, the large footprint of UEAs causes extensive FBR, bleeding and necessity to use pneumatic inserters. Micromachined electrodes generally do not corrode or produce toxic species and their electrical properties can be largely tuned by application of extra layers of materials, but often suffer from delamination problems.

Polymer technology could bring an answer to the problem of forming highly customized, repeatable, three-dimensional electrodes as they offer possibilities to be cast, printed, moulded, and functionalized like no other material. Moreover, their flexibility, which is advantageous from FBR point of view, causes serious implantation problems. Nonetheless, poor long-term *in-vivo* stability of polymeric materials precludes their domination in the chronic neural recordings field in the near future.

It can be also observed that inherently three-dimensional implants (microwires, Utah electrode arrays) perform overall better *in-vivo*. To fulfill the needs of progressively smaller and more complicated microelectrodes use of novel materials and integration of nanotechnology with existing microfabrication strategies could benefit in the realization of the new designs and fabrication standards. Fortunately, miscellaneous fabrication strategies can be combined to create the best possible output.

Questions of limiting body response, finding an ideal set of probe's properties, providing fully autonomous power delivery and high bandwidth communication remain mostly unanswered. Detailed studies on potential influence of manufacturing methods on final material and implant biocompatibility must be conducted. With the current state of knowledge, it is not known whether various chemicals and processes to which arrays are exposed during fabrication do not leave the trace on or alter material's biostability. The mechanisms contributing toward materials degradation, especially for hybrid, composite, and compound materials, are not well-explained. Moreover, testing passive electrodes in small biological models such as rodents might not be translatable to the behavior of active devices implanted in much larger human brain. For that reason, there is still a long way to go until invasive neural interfaces will be reliable enough to allow them to be used in the human applications as a safe treatment or diagnostic devices. Current neural electrodes are implanted in very different ways thus direct comparison of their behavior might be blurred by an influence of the technique and quality of the surgery. This is valid especially for acute studies as it was shown that the most of anesthesia drugs decrease neuronal firing rates up to few days after the surgery, depending on an animal.

Until any device will be commercially available and considered clinically viable, much work needs to be focused on establishing main factors influencing an extent of foreign body reaction. Perhaps instead of trying to limit FBR's, cellular encapsulation could be used to anchor implants to limit relative micro-movements. Initial activation of microglial cells could also be used to the benefit of long–term implantation, as it was shown they could promote restoration of damaged neurons by the presence of neurotropic factors (Eddleston and Mucke, 1993). Much of the trouble with currently available implant geometries results from the majority of studies being focused on highly localized single and multi-unit recordings, which after risk-reward analysis might not be the best option. Shifting the attention onto translation of local field potential-based systems may provide a means for achieving increased chronic-stability. LFP signals are generally easier to observe (i.e., LFP signals are often observable in the absence of single unit activity) and have been shown to contain useful information while not being as affected by changes in local cellular architecture. Future electrodes could additionally be equipped with actuation mechanisms that are activated post-surgically to penetrate through the glial encapsulation after its formation; it has been shown that the impact of any secondary tissue reaction is less adverse.

The focus of scientists of different backgrounds should be brought to cooperatively engineer novel materials and packages, manufacturing techniques, as well as integration and implantation methodologies, which could overcome prolonged stability limitations of existent neural probes. Currently existing materials do not have proper set of mechanical, biological nor electrical properties to match neural tissue, thus creating the need for the hybrid materials that could offer such. It is expected that the future will bring smaller, autonomous, wireless neural probes able to perform decade-long recording and stimulation, while having more channels, and fewer feedthroughs. Assuming a future where neural probes are chronically -stable, safe, and widely accessible, new opportunities would certainly present themselves. Those would be available both to people affected by neural and post-traumatic conditions in the form of electroceutical-treatment, and for healthy individuals willing to add or enhance their abilities. The potential markets for the future brain-machine interfaces are vast and range from medicine and diagnostics, to consumer electronics and entertainment. Thanks to the number of large, well-funded neurotechnology initiatives worldwide, the community can address the technological grand challenges we have identified through reviewing the present state of the art.

Only then can the true impact of next generation neural interfaces for intracortical recording be realized.

## Author contributions

Drafting of manuscript, study conception and design: KS and TC. Acquisition of data: N/A (review paper). Analysis and interpretation of data, critical revision and final approval of the version to be published: KS, TC, and LG.

### Conflict of interest statement

The authors declare that the research was conducted in the absence of any commercial or financial relationships that could be construed as a potential conflict of interest.

## Footnotes

^1^Technologies, Tucker-Davis. “Omnetics Based Electrodes.” Tdt.Com, Web. 14 Sept. 2016. Available online at: http://www.tdt.com/omnetics-based-electrodes.html.

^2^“Thumbtack Probe.” Thumbtack Probe | Plexon. N.p. Web. 12 Sept. 2016. Available online at: http://www.plexon.com/products/thumbtack-probe.
